# Discrete Relationships between Spatiotemporal Gait Characteristics and Domain-Specific Neuropsychological Performance in Midlife

**DOI:** 10.3390/s24123903

**Published:** 2024-06-17

**Authors:** Laura Morrison, Adam H. Dyer, Helena Dolphin, Isabelle Killane, Nollaig M. Bourke, Matthew Widdowson, Conor P. Woods, James Gibney, Richard B. Reilly, Sean P. Kennelly

**Affiliations:** 1 Tallaght Institute of Memory and Cognition, Tallaght University Hospital, D24 NR0A Dublin, Ireland; 2 Discipline of Medical Gerontology, School of Medicine, Trinity College Dublin, D02 PN40 Dublin, Ireland; 3 Department of Engineering, Technological University Dublin, D07 EWV4 Dublin, Ireland; 4 Department of Clinical Medicine, School of Medicine, Trinity College Dublin, D08 W9RT Dublin, Ireland; 5 Robert Graves Institute of Endocrinology, Tallaght University Hospital, D24 NR0A Dublin, Ireland; 6 Trinity Centre for Biomedical Engineering, Trinity College Dublin, D02 R590 Dublin, Ireland

**Keywords:** gait, neuropsychological, cognition, type 2 diabetes, midlife, dual task, automated walkway, cognitive impairment, dementia

## Abstract

Midlife risk factors such as type 2 diabetes mellitus (T2DM) confer a significantly increased risk of cognitive impairment in later life with executive function, memory, and attention domains often affected first. Spatiotemporal gait characteristics are emerging as important integrative biomarkers of neurocognitive function and of later dementia risk. We examined 24 spatiotemporal gait parameters across five domains of gait previously linked to cognitive function on usual-pace, maximal-pace, and cognitive dual-task gait conditions in 102 middle-aged adults with (57.5 ± 8.0 years; 40% female) and without (57.0 ± 8.3 years; 62.1% female) T2DM. Neurocognitive function was measured using a neuropsychological assessment battery. T2DM was associated with significant changes in gait phases and rhythm domains at usual pace, and greater gait variability observed during maximal pace and dual tasks. In the overall cohort, both the gait pace and rhythm domains were associated with memory and executive function during usual pace. At maximal pace, gait pace parameters were associated with reaction time and delayed memory. During the cognitive dual task, associations between gait variability and both delayed memory/executive function were observed. Associations persisted following covariate adjustment and did not differ by T2DM status. Principal components analysis identified a consistent association of slower gait pace (step/stride length) and increased gait variability during maximal-pace walking with poorer memory and executive function performance. These data support the use of spatiotemporal gait as an integrative biomarker of neurocognitive function in otherwise healthy middle-aged individuals and reveal discrete associations between both differing gait tasks and gait domains with domain-specific neuropsychological performance. Employing both maximal-pace and dual-task paradigms may be important in cognitively unimpaired populations with risk factors for later cognitive decline—with the aim of identifying individuals who may benefit from potential preventative interventions.

## 1. Introduction

Midlife cardiovascular risk factors such as type 2 diabetes mellitus (T2DM), obesity, and hypertension confer a significant risk for dementia in later life [1,2,3]. Amongst individuals with these risk factors, identification of those most at risk is an urgent priority to allow potential multi-domain preventative interventions at an early stage [4,5,6]. Optimal identification of those most at risk at a population level may identify those who may benefit most from such targeted approaches.

An important integrative biomarker of neurocognitive function across the lifespan is gait speed and spatiotemporal gait characteristics [7]. Gait requires integrity of both musculoskeletal and central nervous systems, and its measurement appears to be a useful proxy for health and physical function [8,9]. Importantly, a decline in gait speed is associated with the development of dementia in older adults [10,11,12,13,14,15]. Additionally, spatiotemporal gait characteristics, reflecting different domains of gait including pace (stride and step lengths), rhythm (swing, step, stance, and stride times), and variability (standard deviations of step/stride length and time), are associated with both cognitive decline and dementia [16].

An important factor to consider in assessing relationships between gait characteristics and neurocognitive function is the specific gait task or paradigm. Increasingly, studies show that the addition of a cognitive task (cognitive “dual-task”) may uncover associations not seen by measuring single-task gait speed, reflecting greater ecological validity given the ubiquitous nature of multi-tasking in our daily lives [17,18]. In addition to a strong relationship between dual-task performance and executive function, studies have also reported associations with short-term memory, processing speed, and attention [8,17,19,20,21]. Given that executive function is one of the first domains affected in individuals with T2DM and other cardiovascular risk factors, the incorporation of dual-task conditions into gait assessments may be beneficial in the identification of those most at risk. [22,23]. Apart from introducing an additional task to single-task paradigms, assessing maximal gait speed (and not just usual gait speed) may also reveal differing associations with neurocognitive function in older adults—and potentially may have a role in probing executive function in particular [24,25].

Most studies examining links between spatiotemporal gait characteristics and cognitive function have been performed exclusively in older adults. A notable exception is the Dunedin study, which has reported novel associations between slower gait speeds on single/dual task and neurocognitive function not only in midlife, but across the lifespan [26]. Further, in this study those with slower gait speeds in midlife also had greater accumulation of white matter hyperintensities, more cortical thinning, and smaller cortical surface area on neuroimaging in addition to accelerated biological ageing across multiple systems [26]. Recently, a study from the Barcelona Brain Health Initiative on middle-aged individuals has reported that dual-task gait begins to deteriorate in the 6th decade of life, a change explained by inter-individual variance in cognition [27]. Taken together, these studies indicate that gait is closely related to neurocognitive function across the lifespan.

Given the urgent need to identify those in midlife at greatest risk of later dementia, we sought to assess the complex relationships between a broad number of spatiotemporal gait characteristics and domain-specific neuropsychological performance in middle-aged adults with and without T2DM—a significant risk factor for later dementia. We hypothesised that analysis of spatiotemporal gait performance across three tasks (usual pace, fast pace, and cognitive dual task) and detailed neuropsychological performance would reveal discrete and differing correlations between spatiotemporal gait characteristics and cognitive function. This may inform ongoing efforts at real-world validation and the utility of gait as a marker of neurocognitive function in individuals at risk for the later development of dementia in midlife.

## 2. Materials and Methods

### 2.1. Setting and Participants

Cross-sectional data for the current study comes from the second wave of the Exploring Novel Biomarkers of Brain Health in Type 2 Diabetes (ENBIND) study. ENBIND is a longitudinal study based in Dublin, Ireland, that aims to explore the potential biomarkers of cognitive function in middle-aged individuals with and without T2DM to potentially identify those at greater risk of later cognitive decline [28,29,30]. In this study, participants are selected to represent otherwise healthy middle-aged individuals free from any significant medical comorbidity apart from uncomplicated T2DM. We assessed those free from any objective T2DM-related complications to assess the earliest possible stages in the link between gait and cognitive function in uncomplicated T2DM.

Participants with T2DM consisted of otherwise healthy, cognitively unimpaired middle-aged individuals (aged 35–65) with no established microvascular or macrovascular complications recruited from local T2DM clinics. A convenience sample of potential participant’s medical records were screened by a study physician at the time of a clinic visit and individuals were approached by a study physician to participate. Age-matched healthy controls (HC) were recruited by local advertisement in a 2:1 T2DM:HC ratio. Ethical approval was granted from the institutional ethics committee and the research was conducted in accordance with the declaration of Helsinki.

Of note, individuals were excluded from participation if they had an established diagnosis of cognitive impairment for any reason or known T2DM-related complications (stroke, myocardial infarction, ischaemic heart disease, peripheral vascular disease, retinopathy, peripheral neuropathy, or nephropathy) as per self-report or on chart review of their medical notes. Potential participants with active depression (within the past 6 months), any Diagnostic and Statistical Manual of Mental Disorders Axis I disorder, or existing neurological disorder, significant musculoskeletal, cardiac, or respiratory comorbidity were all excluded from participation to ensure sampling of an otherwise healthy cohort.

### 2.2. Clinical Assessment

All participants underwent a medical review including assessment of medical comorbidity, medications, and questionnaires on risk factors for cognitive decline including family history, alcohol use, and smoking status as well as regular physical activity. The Charlson comorbidity index was used to assess medical comorbidity. Of note, uncomplicated T2DM is included in the Charlson Comorbidity Index. Clinical examination was performed, and the Diabetic Neuropathy Symptom Score (DNSS) administered to identify potential participants with early symptoms of diabetic neuropathy deeming them ineligible for inclusion [31]. All individuals were administered the Montreal Cognitive Assessment as a general cognitive screener. Any individual scoring less than 23 was excluded from participation as this cut-off may indicate potential impairment based on normative data from the Irish population [32]. Additionally, the Centre for Epidemiological Studies Depression Scale was administered and those scoring greater than 10 were excluded—indicative of potentially significant current depressive symptoms, again in line with previously published Irish representative data [33].

Blood pressure was measured in the seated position using an automated sphygmomanometer after a 5 min rest and a non-fasting blood draw was obtained for lipid profile and Haemoglobin A1c (HbA1c) to assess individuals with T2DM for overall control and to assess HCs for the potential presence of undiagnosed T2DM. A brief T2DM history was also obtained from those with T2DM including years since diagnosis and current medications.

### 2.3. Gait Assessment and Extraction of Spatiotemporal Gait Characteristics

Gait was assessed using a 4.88 m automated walkway (GAITRite, CIR Systems Inc., New York, NY, USA) which has embedded pressure sensors and which acquires gait parameters. GAITRite has been extensively validated and offers detailed spatiotemporal assessment of gait characteristics [34,35]. Assessments occurred in a dedicated gait lab with ceiling lighting and blinds drawn on the room windows. All individuals were provided with identical instructions to complete three gait tasks. The mat was situated longitudinally in the gait lab, with 1.5 m allowed at either end of the mat to enable acceleration and deceleration.

For the first task, the participants were instructed to walk at their usual pace (usual or normal pace condition). For the second task, the participants were instructed to briskly walk at their fastest pace possible—without running (maximal or fast-pace condition). For the third task (cognitive dual task), individuals were instructed to walk at their normal pace, but at the same time perform a cognitive task (serial 7s, starting from the number 96). In between the second and third tasks, individuals were allowed a practice trial of the cognitive task (the same serial 7 task commencing with the number 100). To assess the impact of the addition of a cognitive dual task, the dual task cost (DTC) was computed for each variable which involved subtracting the usual task gait parameter result from the dual-task gait parameter result to obtain the dual-task difference, with this difference expressed as a proportion (percentage) of the usual pace result.

We selected 24 automatically generated gait parameters across five gait domains based on a published report from a large population-based study which assessed the relationships between spatiotemporal gait characteristics and cognitive decline obtained automatically on the GAITRite platform [16]. A total of 24 parameters were extracted for analysis in the current study to capture the broadest possible assessment of gait and identify the most important associations between spatiotemporal gait characteristics and neuropsychological performance in this cohort.

Briefly, the 24 parameters identified from previous reports reflected five gait domains:

(i) Pace: velocity (centimetres/second), stride length (centimetres), and step length (centimetres);
(ii) Rhythm: single-support time (seconds), swing time (seconds), step time (seconds), stance time (seconds), stride time (seconds), and cadence (steps/minute);
(iii) Variability: Stride standard deviation (SD), step length SD, stride velocity SD, stride SD, step SD, swing SD, and stance SD, single-support SD and double-support time SD;
(iv) Phases: single-support time as a % of gait cycle (percentage), double-support time (both values in seconds and percentage of gait cycle), swing time as a % of gait cycle (percentage) and stance time as a % of gait cycle (percentage);
(v) Support: measured primarily as stride width (centimetres).

As measurements between right and left legs showed significant agreement across all parameters, we analysed data from the right leg only, consistent with previous reports using this approach [36,37].

### 2.4. Neuropsychological Assessment

Neuropsychological assessment was performed following an identical protocol for all participants in the same room as the gait lab at the same study visit. Individuals completed a custom-designed battery of tests from the Cambridge Neuropsychological Test Battery (CANTAB) platform specifically designed to probe working/spatial/delayed memory and executive function with all participants receiving the same directions from the tablet computer platform [38,39,40,41]. Testing lasted 60–70 min and included the following tasks: (i) paired-associates learning (PAL)—memorising geometric patterns on screen and results were given as first-attempt memory score (PAL-FAMS; range 0–20); (ii) spatial working memory (SWM)—memorising locations on screen to uncover hidden tokens with results as strategy score (SWMS; range 2–12); (iii) pattern recognition memory (PRM)—recall of specific geometric patterns after a 20 min delay scored as percentage correct delayed (PRMPCD; range 0–100%); (iv) reaction time task–reaction time to circles on screen in ms; (v) rapid visual processing (RVP)—detecting sequences of numbers amongst a rapidly changing series of digits measured as a signal detection or “A prime” measure (RVPA; range 0.00–1.00); and (vi) one-touch stockings of Cambridge (OTS)—matching patterns by moving coloured balls inside stockings in the minimum number of moves assessed as “problems solved on first choice” (OTSPSFC; range 0–15). These tests were selected to assess immediate and delayed memory and the attention and executive function domains, which are amongst the earliest affected in T2DM-related cognitive dysfunction [22,23].

### 2.5. Statistical Analysis

Baseline characteristics between those with and without T2DM are summarised using means and standard deviations, medians with interquartile ranges, or proportions where appropriate. Univariate comparisons of gait characteristics in those with and without midlife T2DM were performed using *t*-tests or Wilcoxon rank-sum tests as appropriate. Data from both neuropsychological and spatiotemporal gait assessment parameters were inspected using histograms and Q-q plots for normality. All neuropsychological variables were normally distributed. The distribution of normality for the spatiotemporal gait characteristic varied by each parameter and so non-parametric analyses were used to compare between-group differences (T2DM vs. HC). To aid in further assessment and interpretation, z-scores were created for each neuropsychological and spatiotemporal gait variable in the overall cohort.

To assess the relationship between each gait parameter across the three walks and cognitive function, linear regression was used with neuropsychological test z-score as the dependent variable and gait parameter z-score as the independent (predictor) variable. Associations were first tested unadjusted (Model 1; unadjusted), with subsequent adjustment for age, sex, education status, body mass index (BMI), and T2DM status (Model 2; adjusted). Further, as we analysed the results of the overall cohort, we subsequently assessed the interaction between T2DM status and each gait parameter as the independent variable of interest (T2DM*gait parameter interaction term) to examine for any potential T2DM-specific associations.

To reduce the number of variables explaining variability in gait performance across the cohort, we used principal components analysis (PCA) as a dimensionality-reduction method. This enabled us to use a smaller number of variables to retain as much of the variability as possible in the larger dataset. To inspect data for PCA suitability, we used the Kaiser–Meyer–Olkin measure and the Bartlett test of sphericity. This confirmed the suitability of the gait dataset for analysis using a PCA approach. A PCA analysis was performed for the 24 gait parameters for each walking task separately, with components scored for each walk. We then took the first three components of the PCA with the greatest eigenvalues to examine their association with scores on each cognitive test. The results are reported for each component, the proportion (in percentage) of variance explained, and the corresponding eigenvalue.

To examine the relationship between PCA components and cognitive performance, linear regression was used with neuropsychological test Z-score as the dependent variable and each principal component as the independent variable and using the same model adjustment as above. Associations were tested in the first instance across the entire cohort (T2DM and HC, as above). Again, we assessed for any T2DM-specific interactions using a T2DM*principal component interaction term using the same model adjustment. All data analysis and visualisation were conducted in STATAv17.0 (Statacorp, College Station, TX, USA), and RStudio with an alpha level of *p* < 0.05 considered statistically significant.

## 3. Results

### 3.1. Cohort Details and Neuropsychological Performance

Overall, 102 individuals meeting the above inclusion criteria were assessed including 65 individuals with uncomplicated T2DM (aged 57.5 ± 8.0 years; 26/65, 40% female) and 37 HC (aged 57.0 ± 8.3 years; 23/37 62.1%). On comparing both groups, there was a significantly greater proportion of females in the HC group vs. those with T2DM, whilst those with T2DM had a significantly higher BMI, burden of polypharmacy, comorbidity index, and lower total cholesterol and low-density lipoprotein levels (all *p* < 0.00, Table 1). There were no differences between the groups in terms of smoking status, alcohol use or physical activity. Detailed characteristics of both groups are provided in Table 1 below. Neuropsychological performance was largely similar between those with and without T2DM. However, T2DM was associated with small but significantly decrements in reaction time (z = −2.01, *p* = 0.04) and rapid visual processing (z = 2.05, *p* = 0.04). The results of neuropsychological assessments are provided in Table 1 below.

### 3.2. Gait Characteristics in Individuals with Midlife Type 2 Diabetes Mellitus

**Figure 1:**
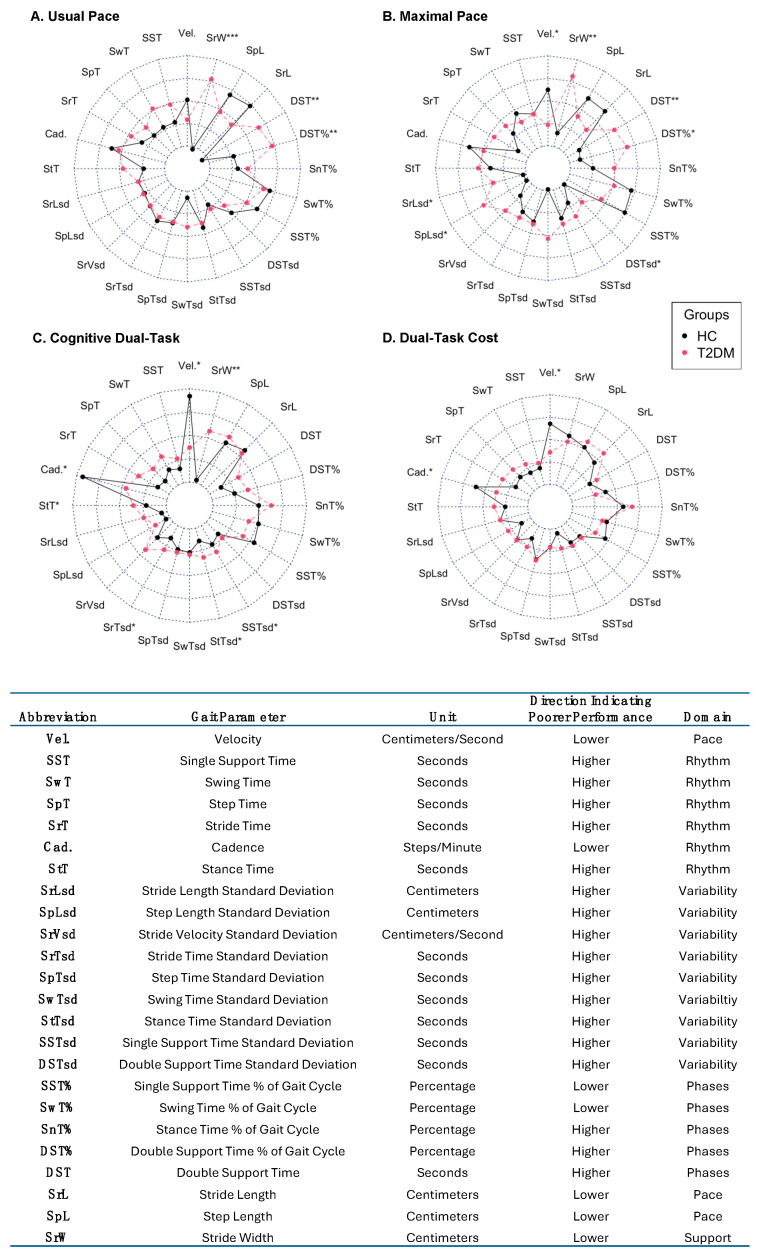
Spatiotemporal Gait Characteristics by T2DM Status. Participants were instructed to walk at their “usual” (or “normal”) pace, “maximal” (or “fast”) pace and again at their normal pace with the addition of a cognitive dual task (serial 7s). Following previous approaches, 24 spatiotemporal gait characteristics were extracted from the GaitRite™ automatic walkway. Radar plots illustrate the median z-score for each domain by group. (**A**–**D**) Radar plots illustrate the median value as a z-score for T2DM and HC groups. Differences between groups were assessed using Wilcoxon rank-sum tests. (Supplementary Table S1). * *p* < 0.05, ** *p* < 0.01, *** *p* < 0.001.

In order to understand the impact of type 2 diabetes mellitus (T2DM) on spatiotemporal gait characteristics, each of the 24 parameters were compared between T2DM and HC across the three different walk conditions individually. Results are visualised in Figure 1 indicating those parameters which significantly differed between groups and are provided in full in Supplementary Table S1. On the usual pace walk, those with T2DM had a greater double-support time and a greater stride width (all *p* < 0.05, see Supplementary Table S1). On the maximal-pace walk, small but statistically significant differences were observed for a slower gait speed and greater stride length variability (SD: standard deviation), step length variability (SD) and double-support time standard deviation in those with T2DM. Similar to the usual pace task, T2DM was associated with longer double-support time and stride width, with some additional contributions from variability parameters (all *p* < 0.05, Supplementary Table S1, Figure 1). For the cognitive dual-task condition, T2DM was associated with a slower velocity and cadence, greater stride width as well as longer stride time, stance time, and single-support time standard deviations (all *p* < 0.05, Supplementary Table S1, Figure 1). For dual-task cost, differences were only observed for velocity and cadence. The results for all parameters are represented in Figure 1 and Supplementary Table S1. Thus, midlife T2DM was associated with statistically significant differences in phases and support gait domains for the usual-pace walk, and additionally with variability, pace, and rhythm for the fast walk, additionally with gait variability for the cognitive dual task. Figure 1 demonstrates the median score per group (HC vs. DM) for all parameters and indicates that those parameters significantly differ between those with DM and HC (Wilcoxon rank-sum test, * *p* < 0.05, Figure 1).

### 3.3. Relationships between Gait Prameters and Neuropsychological Performance

Given that T2DM was only associated with only small neuropsychological decrements, we analysed relationships between spatiotemporal gait characteristics and neuropsychological performance in the overall cohort (T2DM and HC combined), followed by analysis for any T2DM-specific associations. For the usual pace task, associations were seen between quicker gait velocity and better performance on immediate memory performance (adjusted *p* < 0.05, Supplementary Table S2). Similarly, longer step time and slower cadence were associated with poorer working memory performance (adjusted *p* < 0.05, Supplementary Table S2). Greater stride length and width were both associated with poorer working memory performance (adjusted *p* < 0.05, Supplementary Table S2). Greater stride length was associated with better reaction times and executive function performance (adjusted *p* < 0.05). Thus, during the usual pace condition, the gait pace and rhythm domain parameters were associated with working/delayed memory, with consistent associations between greater stride length and poorer memory/executive function and reaction time performance.

On the maximal-pace walk, longer single-support, swing, step, and stride times and a quicker cadence (steps/minute) were associated with quicker reaction times and better delayed memory performance (adjusted *p* < 0.05, Supplementary Table S2). Stride length, step length, and step width were associated with working memory performance during the maximal pace task, with greater stride length associated with better performance on the immediate memory task (adjusted *p* < 0.05, Supplementary Table S2). Greater stride length was associated with better executive function performance. Taken together, the analysis demonstrates that the maximal-pace task revealed associations between additional pace (e.g., stride/step/swing times) domain parameters and both delayed memory and executive function/reaction time performance.

On the addition of a cognitive dual task, differing patterns of associations were observed. Greater stride/stance times and greater variability in terms of their standard deviations were associated with poorer delayed memory performance (*p* < 0.05, Supplementary Table S2). Longer swing time and stance time as a % of the gait cycle were associated with better and poorer performance on the immediate memory task. Greater double-support time was associated with poorer delayed memory performance and greater stride length with better delayed memory performance (adjusted *p* < 0.05, Supplementary Table S2). Greater velocity during the dual-task paradigm was associated with better performance on both executive function tests (RVP/OTSC). Greater stride length in the cognitive dual task was associated with greater reaction times and better performance on both executive function tasks (all adjusted *p* < 0.05, Supplementary Table S2). Taken together, there is a consistent contribution from pace and rhythm (as seen in the usual pace walk) with memory and executive function performance as well as notable associations between variability (most consistently stance time standard deviation) and memory executive function performance not seen in single-task conditions. All of the results are given in tabular format in Supplementary Table S2, both unadjusted (Model 1) and with covariate adjustment (Model 2).

Several of these associations differed slightly when analysing cognitive dual-task cost. Increasing cost in terms of stride time, stance time, step length, and time standard deviations, single-support and double support times were associated with poorer delayed memory performance (all adjusted *p* < 0.05, Supplementary Table S2). Greater step length and stride length cost were both associated with better performance on both tests of executive function (adjusted *p* < 0.05, Supplementary Table S2). Analysis of the dual-task cost highlights relationships between greater dual-task cost variability (in terms of standard deviations of step time and step length) and delayed memory performance. For all tasks, all analyses were repeated using a T2DM*gait parameter interaction term as the independent variable of interest; however, this did not reveal any T2DM-specific associations between gait parameters and cognitive function.

### 3.4. Principal Components Analysis of Gait Performance

**Figure 2:**
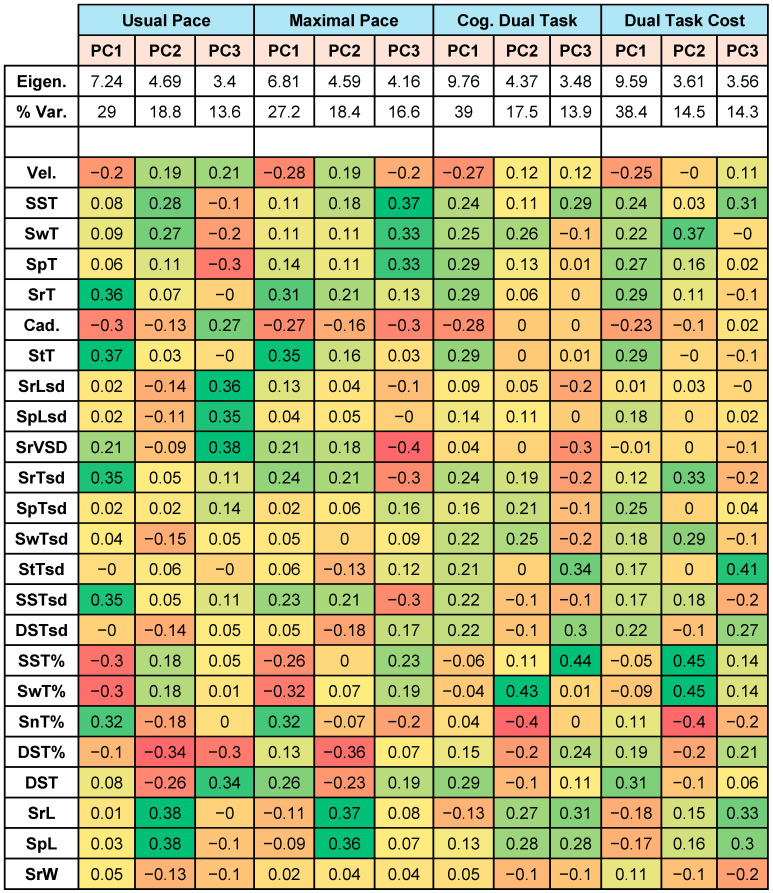
Principal Components Analysis of Spatiotemporal Gait Characteristics Across Usual-Pace, Maximal-Pace, and Cognitive Dual-Task Conditions. Principal components analysis (PCA) was performed separately for each walk—usual pace, maximal pace, cognitive dual-task pace, and dual-task cost for each parameter computed. The first three PCA components explained 61.4%, 62.2%, and 70.4% of the variance across the three tasks and 67.1% of the variance in dual-task cost. Eigenvalues and % variance explained for each component are listed above a breakdown of gait parameters contributing to each component. Full names for each gait domain acronym are provided above in Figure 1. Data are coloured from red (indicating the strongest negative contributions within each PCA) to green (indicating the strongest positive contributions within each PCA). PC1: Principal Component 1; PC2: Principal Component 2; PC3: Principal Component 3; Cog.: cognitive; Eigen.: eigenvalue; Var. %: variance % explained.

Given the number of associations between spatiotemporal gait characteristics and neuropsychological function, we used dimensionality reduction methods to reduce the number of explanatory gait variables and highlight key domains of gait associated with neuropsychological performance. By using PCA independently on each gait task, principal components were defined to explain variability in gait performance. We examined the first three unrotated components in detail given they explained the majority of the variance across each task in the overall cohort. The first three principal components derived from each separate task explained 61.4% of the variance in the usual-pace walk, 62.2% of the difference in maximal-pace walking, and 70.4% of the variance in the cognitive dual-task walk (and 67.1% of the dual-task cost). The principal components, their eigenvalues, their contribution to variance, and the contributing gait parameters are given in Figure 2.

PC1 across each gait task was somewhat similar across all walks—with strong contributions from velocity and cadence, stride and stance time, stride time/single-support time standard deviations and the % of gait cycle spent in stance, single-support, and swing time. PC1 in the dual-task paradigm had greater contributions from swing, step, stride, and single-support times and their standard deviations. PC2 had greatest contributions from step and stride length, swing and step time with strong negative contributions from double-support time. For the dual task for both PC1 and PC2, the contributions of the measures of gait variability were higher for standard deviations of stride, step, and swing time. PC3 showed more inconsistency across tasks, with PC3 on the usual-pace walk demonstrating strong contributions from standard deviations of stride and step length as well as stride velocity. For the maximal-pace walk, PC3 had strong contributions from single-support time, swing time, and step time whilst for the dual-cognitive task, the strongest contributions were seen from stride length, step length, single-support time as a percentage of gait cycle and stance time standard deviation.

### 3.5. Associations between PCA Components and Neuropsychological Performance

**Figure 3:**
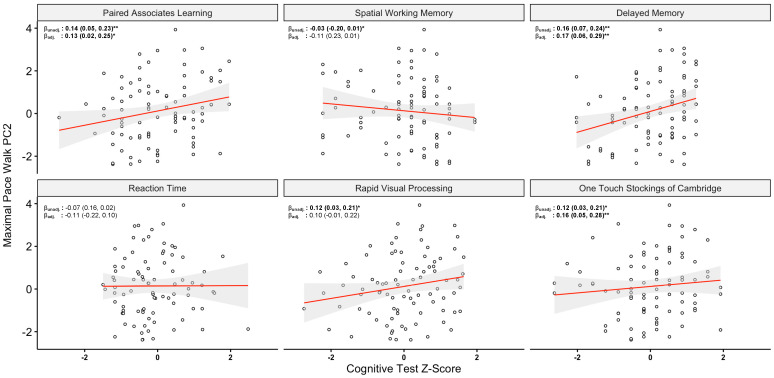
Association Between Maximal Pace Principal Component 2 and Neuropsychological Performance. Scatterplots demonstrate the association between maximal-pace PC2 score and z-scored performance on each neuropsychological test. Linear regression results as given in Table 2 above are provided for unadjusted and adjusted models. In the unadjusted model, PC2 was the independent variable and neuropsychological test z-score the dependent variable. The adjusted model applied adjustment for age, sex, body mass index, education and type 2 diabetes mellitus status. The results are indicated in the top left of each graph and presented as beta coefficients (β) with corresponding 95% confidence intervals (95% CIs). * *p* < 0.05, ** *p* < 0.01.

To assess which of the above composite components were most associated with cognitive performance, we employed linear regression to assess relationships between principal components (independent variable) and cognitive score (dependent variable) both in unadjusted and fully adjusted models. The full results of this analysis are given below in Table 2. The most consistent association observed was for PC2 on the maximal-gait task and poorer performance across nearly all tasks. Under fully adjusted models, greater PC2 values were associated with both better immediate memory (β: 0.13, 0.02–0.25; *p* < 0.01) and delayed memory (β: 0.17, 0.06–0.29; *p* < 0.05) performance as well as better executive function as assessed (β: 0.16, 0.05–0.28; *p* < 0.01). Both an association between better performance on rapid visual processing and maximal-gait PC2 in addition to a significant association between increasing PC2 and poorer performance on spatial working memory did not persist on covariate adjustment. This component reflected contributions from the phases (double-support time, single-support time), pace (stride length, step length), and variability domains. Given the significant associations between PC2 and cognitive function, the results are also presented graphically for PC2 and cognitive performance in Figure 3.

## 4. Discussion

In this study, we investigated a broad range of spatiotemporal gait characteristics covering five domains of gait across three different walking conditions in middle-aged individuals with and without risk factors for later cognitive decline/dementia. We found that T2DM was associated with subtle but statistically significant changes in gait—namely slower pace and greater variability. In the overall cohort, we report relationships between spatiotemporal gait characteristics and both memory and executive function in midlife—independent of T2DM status. Importantly, these associations varied by task (single or dual task) and condition (slow or fast walk). These results suggest that spatiotemporal gait characteristics may be important integrative biomarkers reflecting central nervous system functioning and may have utility in midlife to act as markers for cognitive function. This is particularly pertinent given that executive function and memory domains are those that may be first affected in individuals with T2DM.

Our findings regarding the impact of T2DM on gait function are somewhat consistent with the previous literature. However, it is important to note that we included individuals without any complications of T2DM, with good cardiovascular primary prevention, and all individuals were screened for peripheral neuropathy. This is an important strength of our study in aiming to assess the earliest possible signs of the relationship between gait and neurocognitive function in individuals both with and without risk factors for later cognitive function. Previous studies have demonstrated shorter pace domain parameters in older adults with T2DM [37]. There is also some evidence that T2DM may be associated with slower gait velocity, shorter stride length, and longer stride time—although most prior studies included individuals with peripheral neuropathy, a potential significant confounder when assessing gait characteristics from neurocognitive perspective [42]. The effect sizes of the current study are somewhat smaller and may indicate the earliest possible signs of this relationship. Reflecting the complexity of the various physiological contributions to gait performance, a small study which also demonstrated increasing gait variability in T2DM on cognitive dual tasking in older adults found the association did not differ by the presence of neuropathy [43]. Importantly, one of the key strengths of our study is that we purposefully recruited an otherwise healthy middle-aged cohort of individuals with T2DM free from cognitive impairment and screened for peripheral neuropathy.

In interpreting our findings, it is worthy of discussion that the effect sizes observed were small. We observed very small differences in cognitive function between T2DM and HCs and these are unlikely to be of clinical significance in the context of previous reports [38,39,40,41]. Again, when assessing differences in gait performance between T2DM and HCs, the differences were small and of unclear clinical significance. Thus, our findings largely pertain to a healthy midlife cohort, some of whom have risk factors for later cognitive decline—but at the exact window when these risk factors may be exerting their determinate influence. This is one of the first studies to demonstrate discrete relationships between spatiotemporal gait performance and neuropsychological performance in otherwise healthy individuals with risk factors for cognitive decline. Longitudinal studies—including longitudinal assessment of the current cohort—are crucial in identifying whether these small decrements do indeed identify individuals at greatest risk of later cognitive decline.

We noted differing patterns of association by gait task when assessing relationships between spatiotemporal characteristics/domains of gait and cognitive function. Our findings linking usual pace walking gait pace and rhythm domains with memory and executive function are consistent with the previous literature [44,45,46]. Our results for the additional associations seen on the maximum-speed gait linking rhythm, pace and variability domains with cognitive function expand upon previous reports and suggest inclusion of this condition in future studies of midlife risk [24,25,47]. Maximal gait may probe specific associations between spatiotemporal gait and cognitive function in midlife, although it is important to remember that this may also be a proxy for cardiorespiratory fitness and musculoskeletal function. Importantly, our findings linking these tasks and cognitive function did not differ by T2DM status and most persisted in models adjusting for T2DM. Thus, they may indeed reflect shared underlying neurocognitive correlates between gait function and neuropsychological performance independent of T2DM status per se.

When studying dual-task gait, we replicate previous work in older adults suggesting that dual-tasking variability is related to cognitive function [48]. Interestingly, when we view our results on dual tasking in the context of the Barcelona Brain Health Initiative, the included age of patients in the present report maps very closely onto the age at which dual tasking begins to diverge [27]. In the Barcelona study, stride time and its variability during cognitive dual-task gait began to change between 54 and 57 years and the variance was largely due to cognitive function. Thus, the data in the current study linking gait variability during dual tasking may represent early signs of cognitive impairment; however, only longitudinal follow-up of this and other cohorts will determine if this is the case. Nevertheless, our data underscores the importance of measuring cognitive dual-task and not just single-task gait in midlife studies.

Our study has several notable strengths. We comprehensively assessed gait using an automated runway, commonly considered the gold standard in spatiotemporal gait assessment [49]. This allowed us to extract a relatively large number of spatiotemporal gait characteristics in comparison to other studies. These parameters are not typically available using manual or other gait paradigms. As the field moves forward, given the clear constraints of using automated walkways in terms of physical space, requirement for support and maintenance and cost, it is likely that inertial motion unit (IMU)- and video-based assessment of gait using mobile and other applications will become increasingly useful [50]. As they do, our results advocate for the inclusion of multiple gait tasks with multiple parameters spanning different domains of gait, as early associations—as in the current study—may be observed from parameters above and beyond gait speed or velocity alone.

Another key strength of our study is also notable in that it was a healthy midlife cohort and adds important insight into the early relationships between decrements in spatiotemporal gait performance and neurocognitive decline in midlife. This reflects previous findings in midlife which has demonstrated relationships between gait and neurocognitive performance. Finally, our study is also notable for the comprehensive neuropsychological assessment battery that was performed, allowing more granular analysis of cognitive function then may be obtained from general cognitive screeners alone. This approach allowed us to assess domain-specific cognitive performance, importantly including those domains first affected by individuals with midlife T2DM.

There are also several important limitations in the current study. Our study was cross-sectional in nature as the automated runway to assess gait was only incorporated in the second wave of the ENBIND study. Thus, our findings are limited to cross-sectional data analysis. Longitudinal analysis of this and other cohorts will help determine the precise longitudinal relationships between spatiotemporal gait across differing tasks and subtle neurocognitive decline in midlife. Understanding longitudinal relationships between small decrements in gait performance and neuropsychological performance will be crucial in the validation of gait as an integrative marker of neurocognitive decline as the field moves forward. Similarly, our study was relatively small by comparison to other studies in the literature and may be underpowered to detect some of the more subtle associations linking spatiotemporal gait and neuropsychological performance. Our study was a single-site study with a homogenous participant population and the applicability of these findings to different clinical and other cohorts may be somewhat limited. It is also worth considered confounding factors which may influence gait performance in T2DM and midlife risk factors for cognitive decline. Medical comorbidity, medication use, fitness, BMI, and other factors may exert important influences. However, it is important to note that our findings persisted for covariate adjustment including age, sex, education, and BMI. Finally, our study only measured walking bouts of 4 m which may be too short to obtain a full assessment of gait variability. However, notwithstanding this, we demonstrated significant associations between gait variability and cognitive function on both maximal speed and cognitive dual-task paradigms.

## 5. Conclusions

In conclusion, we demonstrated discrete relationships between spatiotemporal gait characteristics across three specific gait conditions and detailed neuropsychological function in middle-aged adults, some of whom have risk factors for later cognitive decline. Our data suggests that assessing for gait in midlife may reveal subtle early signs of neurocognitive dysfunction and that gait is an important integrative marker of overall neurocognitive function. We observed different associations between gait characteristics and cognitive performance depending on task, but these did not differ by T2DM status. We suggest including both maximal and dual-task gait conditions when assessing links with cognitive function as these may reveal associations not seen with the most common method of gait assessment in such studies—namely single-task, usual-pace gait walks.

## Figures and Tables

**Table 1:** Characteristics of ENBIND Study Participants, by Type 2 Diabetes Mellitus (T2DM) Status.

Characteristic	HC (*n* = 37)	T2DM (*n* = 65)	Statistic
Age, years (SD)	57.0 (8.3)	57.5 (8.0)	t = −0.31, 0.62
Sex, female (*n*, %)	23 (62.1%)	26 (40.0%)	*χ*^2^ = 4.64, *p* = 0.03
Body Mass Index, kg/m^2^ (SD)	28.4 (3.5)	31.6 (6.8)	t = −2.74, *p* = 0.003
Education			
*Primary (n, %)*	2 (5.4%)	8 (12.3%)	
*Secondary (n, %)*	24 (64.9%)	46 (70.8%)	
*Tertiary (n, %)*	11 (29.7%)	11 (16.9%)	*χ*^2^ = 3.06, *p* = 0.22
Depressive Symptoms, CESD-D (IQR)	4 (1–6)	6 (3.5–7)	z = −1.71, *p* = 0.09
Polypharmacy, >5 medications (*n*, %)	2 (5.4%)	36 (55.4%)	*χ*^2^ = 25.2, *p* < 0.001
Blood Pressure			
*Systolic, mmHg (SD)*	136.7 (14.0)	135.2 (14.2%)	t = 0.53, *p* = 0.70
*Diastolic, mmHg (SD)*	81.2 (8.6)	80.0 (9.0%)	t = 0.70, *p* = 0.24
Lipid Levels			
*Low-Density Lipoprotein (SD)*	2.9 (1.2)	2.2 (1.0)	t = 2.91, *p* = 0.002
*Total Cholesterol (SD)*	5.0 (1.4)	4.2 (1.0)	t = 3.42, *p* < 0.001
*Triglycerides (SD)*	1.8 (1.7)	2.0 (1.0)	t = −0.70, *p* = 0.76
Smoking Status			
*Never (n, %)*	16 (43.2%)	27 (41.5%)	
*Former (n, %)*	17 (46.0%)	24 (37.0%)	
*Current (n, %)*	4 (10.8%)	14 (21.5%)	*χ*^2^ = 2.03, *p* = 0.36
Alcohol Use			
*Never (n, %)*	11 (29.7%)	11 (16.9%)	
*0–7 Units/Week (n, %)*	11 (29.7%)	33 (50.8%)	
*8–14 Units/Week (n, %)*	10 (27.0%)	13 (20.0%)	
*15–21 Units/Week (n, %)*	4 (10.8%)	4 (6.2%)	
*>21 Units/Week (n, %)*	1 (2.7%)	4 (6.2%)	*χ*^2^ = 5.95, *p* = 0.20
Physical Activity			
*Never (n, %)*	6 (16.2%)	15 (23.1%)	
*Less than Monthly (n, %)*	3 (8.1%)	8 (12.3%)	
*1–3 Times per Month (n, %)*	1 (2.7%)	8 (12.3%)	
*Once per Week (n, %)*	5 (13.5%)	4 (6.2%)	
*2–4 Days Per Week (n, %)*	10 (27.0%)	18 (27.7%)	
*5–7 Days Per Week (n, %)*	12 (32.4%)	12 (18.5%)	*χ*^2^ = 6.80, *p* = 0.24
Charlson Comorbidity Index (IQR)	0 (0–0)	1 (1–1)	z = −7.81, *p* < 0.001
T2DM Characteristics			
*Duration of T2DM, years (IQR)*	-	11 (7–15)	-
*HbA1c (mmol/mmol)*	38.1 (2.9)	60.6 (2.2)	t = −7.7, *p* < 0.001
Medications			
*Number of T2DM Medications*	-	2 (1–3)	-
*Metformin (n, %)*	-	52 (85.3%)	-
*Sulfonylurea (n, %)*	-	14 (23.0%)	-
*GLP-1 Analogue (n, %)*	-	23 (37.7%)	-
*DPP-4 Inhibitor (n, %)*	-	13 (21.3%)	-
*SGLT2 Inhibitor (n, %)*	-	23 (37.7%)	-
*Insulin (n, %)*	-	12 (19.7%)	-
Cognitive Performance			
*Paired Associates Learning, First Attempt (IQR)*	10 (8–14)	11 (7–14)	z = 0.28, *p* = 0.78
*Spatial Working Memory Score (IQR)*	9 (6–11)	9 (7–10)	z = 0.66, *p* = 0.51
*Delayed Pattern Recognition, % Correct (IQR)*	83.3% (72.2–94.4%)	83.3% (72.2–88.9%)	z = 1.55, *p* = 0.12
*Reaction Time Task, ms (IQR)*	390 (359–410)	406 (375–456)	z = −2.01, *p* = 0.04
*Stockings of Cambridge, % Correct (IQR)*	10 (8–11)	10 (8–12)	z = 0.48, *p* = 0.64
*Rapid Visual Processing, A Prime (IQR)*	0.90 (0.87–0.94)	0.88 (0.84–0.92)	z = 2.05, *p* = 0.04

Data are presented as means with standard deviations (SDs) or medians with inter-quartile ranges (IQRs) or numbers (with %) as appropriate. To examine differences between groups, *t*-tests, Wilcoxon rank-sum tests, and chi-square tests were used as appropriate. There were more females amongst HC, whilst individuals with T2DM had a higher BMI, greater polypharmacy, and higher levels of low-density lipoprotein and total cholesterol. Other risk factors such as education levels, smoking, and alcohol use and physical activity levels did not differ between those with and without T2DM. Individuals with T2DM had significantly poorer performance on the Montreal cognitive assessment, as well as a greater reaction time (indicating poorer performance) and poorer performance on the rapid visual processing task. SD: standard deviation; IQR: inter-quartile range; CES-D: center for Epidemiological Studies Depression Scale; T2DM: type 2 diabetes mellitus; HC: healthy controls; HbA1c: Glycated Haemoglobin; GLP-1: Glucagon-Like Peptide 1; DPP-4: Dipeptidyl Peptidase 4; SGLT-2: Sodium-Glucose-Like transporter 2.

**Table 2:** Associations Between Components of Gait and Cognition.

	Paired Associates Learning		Spatial Working Memory		Delayed Pattern Recognition	
	Model 1 β (95% CI)	Model 2 β (95% CI)	Model 1 β (95% CI)	Model 2 β (95% CI)	Model 1 β (95% CI)	Model 2 β (95% CI)
*Usual Pace Walk*						
PC1	−0.01	−0.02	**−0.07**	−0.07	0.04	0.03
	(−0.08, 0.06)	(−0.10, 0.05)	(−0.14, −0.00) *	(−0.14, 0.01)	(−0.03, 0.11)	(−0.05, 0.10)
PC2	0.08	0.09	−0.05	−0.07	0.09	0.10
	(−0.02, 0.17)	(−0.02, 0.21)	(−0.14, 0.04)	(−0.19, 0.05)	(−0.00, 0.18)	(−0.02, 0.22)
PC3	0.05	0.05	0.06	0.03	0.00	0.01
	(−0.06, 0.15)	(−0.06, 0.16)	(−0.05, 0.16)	(−0.08, 0.15)	(−0.11, 0.11)	(−0.10, 0.12)
*Maximal Pace Walk*						
PC1	0.00		0.00	0.01	0.02	0.03
	(−0.07, 0.08)	(−0.06, 0.09)	(−0.07, 0.08)	(−0.07, 0.09)	(−0.05, 0.10)	(−0.04, 0.11)
PC2	**0.14**	**0.13**	**−0.03**	−0.11	**0.16**	**0.17**
	(0.05, 0.23) **	(0.02, 0.25) *	(−0.20, −0.01) *	(−0.23, 0.01)	(0.07, 0.24) **	(0.06, 0.29) **
PC3	-−0.04	−0.03	0.00	0.01	0.06	0.09
	(−0.14, 0.06)	(−0.14, 0.07)	(−0.10, 0.10)	(−0.10, 0.11)	(−0.03, 0.16)	(−0.01, 0.20)
*Cognitive Dual-Task*						
PC1	−0.02	−0.03	−0.01	0.01	**−0.06**	**−0.06**
	(−0.08, 0.05)	(−0.09, 0.03)	(−0.07, 0.06)	(−0.06, 0.07)	(−0.12, 0.01) *	(−0.13, −0.00) *
PC2	**0.11**	**0.10**	−0.06	−0.05	0.09	0.08
	(0.02, 0.20) *	(0.01, 0.20) *	(−0.15, 0.04)	(−0.15, 0.04)	(−0.00, 0.18)	(−0.02, 0.17)
PC3	0.04	0.03	−0.08	−0.07	0.08	0.08
	(−0.07, 0.14)	(−0.07, 0.14)	(−0.18, 0.03)	(−0.18, 0.03)	(−0.03, 0.18)	(−0.03, 0.18)
*Cognitive Dual-Task Cost*						
PC1	−0.02	−0.03	0.01	0.02	−0.06	−0.07
	(−0.09, 0.05)	(−0.10, 0.04)	(−0.05, 0.08)	(−0.04, 0.09)	(−0.13, 0.01)	(−0.14, 0.00)
PC2	0.10	0.08	−0.02	−0.00	0.03	0.01
	(−0.02, 0.22)	(−0.03, 0.19)	(−0.12, 0.08)	(−0.11, 0.10)	(−0.09, 0.15)	(−0.11, 0.13)
PC3	0.02	0.04	−0.09	**−0.10**	0.05	0.07
	(−0.10, 0.14)	(−0.08, 0.15)	(−0.19, 0.01)	(−0.20, −0.01) *	(−0.07, 0.18)	(−0.05, 0.19)
	**Reaction Time Task**		**Rapid Visual Processing**		**Stockings of Cambridge**	
	**Model 1** **β** **(95% CI)**	**Model 2** **β** **(95% CI)**	**Model 1** **β** **(95% CI)**	**Model 2** **β** **(95% CI)**	**Model 1** **β** **(95% CI)**	**Model 2** **β** **(95% CI)**
*Usual Pace Walk*						
PC1	0.02	0.01	0.02	0.00	0.04	0.04
	(−0.05, 0.10)	(−0.07, 0.08)	(−0.06, 0.09)	(−0.07, 0.07)	(−0.03, 0.11)	(−0.04, 0.10)
PC2	−0.06	−0.09	0.05	0.10	0.04	0.10
	(−0.15, 0.03)	(−0.21, 0.02)	(−0.04, 0.14)	(−0.00, 0.22)	(−0.04, 0.13)	(−0.01, 0.22)
PC3	−0.09	−0.04	0.04	0.02	0.05	0.07
	(−0.20, 0.01)	(−0.15, 0.07)	(−0.06, 0.15)	(−0.08, 0.13)	(−0.06, 0.16)	(−0.05, 0.18)
*Maximal Pace Walk*						
PC1	0.07	0.06	−0.01	0.01	0.00	0.00
	(−0.01, 0.14)	(−0.02, 0.13)	(−0.08, 0.07)	(−0.06, 0.08)	(−0.07, 0.08)	(−0.07, 0.07)
PC2	−0.07	−0.11	**0.12**	0.10	**0.12**	**0.16**
	(−0.16, 0.02)	(−0.22, 0.10)	(0.03, 0.21) *	(−0.01, 0.22)	(0.03, 0.21) *	(0.05, 0.28) **
PC3	**0.11**	0.05	−0.05	−0.01	−0.00	0.03
	(0.11, 0.20) *	(−0.05, 0.16)	(−0.15, 0.05)	(−0.10, 0.09)	(−0.10, 0.09)	(−0.07, 0.14)
*Cognitive Dual-Task*						
PC1		0.02	−0.06	**−0.06**	−0.04	−0.05
	(−0.04, 0.09)	(−0.04, 0.09)	(−0.12, 0.01)	(−0.12, −0.00) *	(−0.10, 0.02)	(−0.11, 0.01)
PC2	−0.09	**−0.10**	0.05	0.05	0.07	0.08
	(−0.18, 0.00)	(−0.19, −0.01) *	(−0.04, 0.15)	(−0.04, 0.15)	(−0.03, 0.16)	(−0.02, 0.17)
PC3	−0.02	−0.05	0.08	0.09	0.09	0.10
	(−0.13, 0.08)	(−0.15, 0.05)	(−0.03, 0.18)	(−0.01, 0.19)	(−0.02, 0.19)	(−0.00, 0.20)
*Cognitive Dual-Task Cost*						
PC1	0.01	0.02	−0.05	−0.06	−0.05	−0.04
	(−0.07, 0.09)	(−0.06, 0.10)	(0.13, 0.02)	(−0.13, 0.01)	(−0.11, 0.02)	(−0.06, −0.01)
PC2	−0.06	−0.06	0.01	−0.01	0.02	0.00
	(−0.19, 0.07)	(−0.18, 0.06)	(−0.11, 0.13)	(−0.12, 0.10)	(−0.09, 0.14)	(−0.11, 0.11)
PC3	−0.03	−0.06	0.06	0.09	0.07	0.10
	(−0.16, 0.10)	(−0.18, 0.07)	(−0.06, 0.19)	(−0.02, 0.20)	(−0.04, 0.18)	(−0.01, 0.20)

In order to assess relationships between the composite components detailed above and neuropsychological performance, linear regression was used with the scored component for each condition as the independent variable and the z-score from each cognitive test as the dependent variable. Model 1 presents the results of an unadjusted model whilst model 2 applied adjustment for age, sex, body mass index, education, and type 2 diabetes mellitus status. Data are presented as beta coefficients (β) with corresponding 95% confidence intervals (95% CIs). Consistent associations were seen between the second principal component on the maximal-gait walk and tests of both memory and executive function, some of which persisted following covariate adjustment. PC1: Principal Component 1; PC2: Principal Component 2; PC3: Principal Component 3. * *p* < 0.05, ** *p* < 0.01.

## Data Availability

Research data is not publicly available due to risk of participant identification. Requests for data by suitably qualified researchers can be directed to laura.morrison@tuh.ie.

## Supplementary Materials

The following supporting information can be downloaded at: https://www.mdpi.com/article/10.3390/s24123903/s1, Table S1: Gait Characteristics by Type 2 Diabetes Status; Table S2: Associations Between Gait Domains and Neuropsychological Performance in Middle-Aged Adults with and Without Type 2 Diabetes Mellitus.
