# Supplementary material for: Discrete Relationships between Spatiotemporal Gait Characteristics and Domain-Specific Neuropsychological Performance in Midlife
Source: Sensors (Basel). 2024 Jun 17;24(12):3903. doi: 10.3390/s24123903 (PMC11207228; doi:10.3390/s24123903)
Supplement: Supplementary file 1 [file sensors-24-03903-s001.zip › sensors-3026083-supplementary.pdf]

Supplementary Material

Table S1. Gait Characteristics by Type 2 Diabetes Status

Gait Parameter	HC (n = 37)	T2DM (n = 65)	Statistic
**Usual Pace Walk**			
Velocity, centimetres/second (SD)	117.0 (3.7)	114.5 (1.7)	t = 0.68, p = 0.25
Single Support Time, seconds (SD)	0.39 (0.03)	0.40 (0.04)	t -0.87, p = 0.19
Swing Time, seconds (SD)	0.39 (0.03)	0.40 (0.04)	t = -0.85, p = 0.20
Step Time, seconds (SD)	0.59 (0.21)	0.57 (0.05)	t = 0.96, p = 0.17
Stride Time, seconds (SD)	1.13 (0.32)	1.12 (0.09)	t = 0.51, p = 0.30
Cadence, steps/minute (SD)	105.2 (17.7)	106.6 (8.2)	t = -0.53, p = 0.30
Stance Time, seconds (IQR)	0.68 (0.65, 0.74)	0.72 (0.68, 0.76)	z = -1.43, p = 0.15
Stride Length Standard Deviation, centimetres (IQR)	4.50 (2.32, 6.13)	4.53 (2.71, 7.73)	z = -0.73, p = 0.47
Step Length Standard Deviation, centimetres (IQR)	3.08 (1.92, 4.52)	3.20 (1.86, 4.67)	z = -0.02, p = 0.99
Stride Velocity Standard Deviation, centimetres/second (IQR)	7.02 (4.45, 11.26)	6.94 (3.38, 10.70)	z = 0.68, p = 0.50
Stride Time Standard Deviation, seconds (IQR)	0.04 (0.02, 0.07)	0.03 (0.02, 0.05)	z = 1.53, p = 0.13
Step Time Standard Deviation (IQR)	0.03 (0.02, 0.06)	0.03 (0.02, 0.04)	z = 0.63, p = 0.53
Swing Time Standard Deviation (IQR)	0.01 (0.01, 0.02)	0.01 (0.01, 0.02)	z = 0.56, p = 0.57
Stance Time Standard Deviation (IQR)	0.05 (0.02, 0.06)	0.03 (0.02, 0.05)	z = 1.08, p = 0.28
Single Support Time Standard Deviation (IQR)	0.01 (0.01, 0.02)	0.01 (0.01, 0.02)	z = -0.74, p = 0.46
Double Support Time Standard Deviation (IQR)	0.02 (0.01, 0.03)	0.02 (0.01, 0.04)	z = -0.92, p = 0.36
Single Support Time % of Gait Cycle (IQR)	36.1 (34.6, 37.2)	35.7 (34.7, 36.9)	z = 0.80, p = 0.42
Swing Time % of Gait Cycle (IQR)	36.1 (34.5, 37.4)	35.9 (35, 36.6)	z = 0.68, p = 0.50
Stance Time % of Gait Cycle (IQR)	63.9 (62.6, 65.6)	64.2 (63.4, 65)	z = -0.69, p = 0.49
Double Support Time % of Gait Cycle (SD)	**13.18 (2.20)**	**14.24 (1.86)**	**t = -2.58, p = 0.01**
Double Support Time, seconds (SD)	**0.29 (0.05)**	**0.32 (0.45)**	**t = -2.66, p = 0.01**
Stride Length, centimetres (SD)	135.4 (14.3)	130.7 (16.0)	t = 1.47, p = 0.07
Step Length, centimetres (SD)	67.20 (7.08)	64.82 (8.46)	t = 1.45, p = 0.08
Stride Width, centimetres (SD)	**11.50 (2.73)**	**14.23 (3.58)**	**t = -4.02, p<0.001**

**Maximal Pace Walk**			
Velocity, centimetres/second (SD)	**167.8 (23.5)**	**156.7 (28.1)**	**t = 2.03, p=0.03**
Single Support Time, seconds (SD)	0.35 (0.03)	0.36 (0.04)	t = -0.76, p = 0.22
Swing Time, seconds (SD)	0.35 (0.03)	0.36 (0.04)	t = -0.83, p = 0.80
Step Time, seconds (SD)	0.46 (0.45)	0.48 (0.10)	t = -1.50, p=0.07
Stride Time, seconds (SD)	0.93 (0.13)	0.95 (0.10)	t = -0.72, p = 0.77
Cadence, steps/minute (SD)	129.8 (13.8)	125.8 (15.1)	t = 1.31, p = 0.10
Stance Time, seconds (IQR)	0.58 (0.52, 0.61)	0.59 (0.55, 0.63)	z = -1.64, p=0.10
Stride Length Standard Deviation, centimetres (IQR)	**3.4 (1.41, 5.60)**	**5.36 (2.66, 9.74)**	**z = -2.25, p=0.03**
Step Length Standard Deviation, centimetres (IQR)	**2.67 (2.00, 3.62)**	**4.89 (2.26, 7.56)**	**z = -2.38, p=0.02**
Stride Velocity Standard Deviation, centimetres/second (IQR)	6.64 (5.47, 10.32)	9.30 (4.53, 12.41)	z = -1.20, p=0.23
Stride Time Standard Deviation, seconds (IQR)	0.02 (0.01, 0.04)	0.03 (0.01, 0.05)	z = -0.92, p=0.36
Step Time Standard Deviation (IQR)	0.02 (0.01, 0.03)	0.02 (0.01, 0.03)	z = -0.36, p=0.72
Swing Time Standard Deviation (IQR)	0.01 (0.01, 0.01)	0.01 (0.01, 0.02)	z = -1.66, p=0.10
Stance Time Standard Deviation (IQR)	0.02 (0.01, 0.05)	0.03 (0.01, 0.04)	z = -0.40, p = 0.69
Single Support Time Standard Deviation (IQR)	0.01 (0.01, 0.02)	0.01 (0.01, 0.02)	z = -0.74, p = 0.46
Double Support Time Standard Deviation (IQR)	**0.01 (0.00, 0.02)**	**0.01 (0.01, 0.02)**	**z = -2.01, p=0.04**
Single Support Time % of Gait Cycle (IQR)	38.6 (36.6, 39.8)	37.7 (36.2, 38.9)	z = 1.55, p = 0.12
Swing Time % of Gait Cycle (IQR)	38.5 (37.4, 39.5)	38 (36.7, 39.3)	z = 1.39, p = 0.16
Stance Time % of Gait Cycle (IQR)	61.4 (60.6, 62.6)	62 (60.7, 63.3)	z = -1.45, p = 0.15
Double Support Time % of Gait Cycle (IQR)	**10.7 (9.8,12)**	**11.8 (10.7, 12.8)**	**z = -2.32, p=0.02**
Double Support Time, seconds (SD)	**0.20 (0.04)**	**0.23 (0.05)**	**t = -2.60, p = 0.005**
Stride Length, centimetres (SD)	156.8 (17.6)	151.6 (22.2)	t = 1.23, p=0.11
Step Length, centimetres (SD)	78.45 (8.86)	75.53 (10.87)	t = 1.39, p=0.08
Stride Width, centimetres (SD)	**11.18 (2.16)**	**13.30 (12.37)**	**t = -3.17, p=0.001**
**Dual Task (Cognitive) "Usual Pace" Walk**			
Velocity, centimetres/second (SD)	**97.7 (24.5)**	**87.4 (22.2)**	**t = 2.18, p=0.02**
Single Support Time, seconds (SD)	0.45 (0.14)	0.47 (0.15)	t = -0.92, p = 0.18
Swing Time, seconds (SD)	0.47 (0.22)	0.48 (0.12)	t = -0.35, p=0.63
Step Time, seconds (SD)	0.69 (0.36)	0.71 (0.21)	t = -0.37, p = 0.65
Stride Time, seconds (SD)	1.33 (0.58)	1.36 (0.33)	t = -0.29, p = 0.62
Cadence, steps/minute (SD)	**96.7 (20.0)**	**88.9 (15.9)**	**t = 2.18, p=0.02**

Stance Time, seconds (IQR)	**0.80 (0.74, 0.88)**	**0.84 (0.77, 0.93)**	**z = -2.04, p=0.04**
Stride Length Standard Deviation, centimetres (IQR)	4.68 (2.69, 8.60)	5.82 (4.59, 8.81)	z = -1.55, p=0.12
Step Length Standard Deviation, centimetres (IQR)	3.05 (2.10, 5.03)	3.76 (2.44, 6.09)	z = -1.38, p=0.17
Stride Velocity Standard Deviation, centimetres/second (IQR)	6.36 (3.99, 10.25)	7.23 (4.23, 9.68)	z = -0.14, p = 0.89
Stride Time Standard Deviation, seconds (IQR)	**0.05 (0.03, 0.07)**	**0.08 (0.04, 0.13)**	**z = -2.37, p=0.02**
Step Time Standard Deviation (IQR)	0.03 (0.02, 0.06)	0.04 (0.03, 0.08)	z = -1.38, p=0.17
Swing Time Standard Deviation (IQR)	0.02 (0.01, 0.03)	0.03 (0.01, 0.06)	z = -1.03, p=0.30
Stance Time Standard Deviation (IQR)	**0.04 (0.02, 0.06)**	**0.06 (0.03, 0.09)**	**z = -2.26, p=0.02**
Single Support Time Standard Deviation (IQR)	**0.02 (0.01, 0.03)**	**0.03 (0.01, 0.05)**	**z = -2.33, p = 0.02**
Double Support Time Standard Deviation (IQR)	0.03 (0.01, 0.04)	0.03 (0.02, 0.06)	z = -1.80, p = 0.07
Single Support Time % of Gait Cycle (IQR)	14.4 (12.8, 15.7)	15 (13.8-17.1)	z = -1.73, p = 0.08
Swing Time % of Gait Cycle (IQR)	35.1 (32.6, 36.7)	34.7 (33.1, 36.4)	z = 0.40, p=0.69
Stance Time % of Gait Cycle (IQR)	64.9 (63.3-67.4)	65.4 (63.6, 66.8)	z = -0.40, p=0.68
Double Support Time % of Gait Cycle (IQR)	14.4 (12.8, 15.7)	15 (13.8-17.1)	z = -1.73, p = 0.08
Double Support Time, seconds (SD)	0.42 (0.30)	0.44 (0.15)	t = -0.47, p=0.68
Stride Length, centimetres (SD)	121.1 (15.6)	119.7 (21.9)	t = -0.34, p=0.37
Step Length, centimetres (SD)	60.8 (7.93)	59.6 (10.7)	t = 0.63, p=0.27
Stride Width, centimetres (SD)	**12.39 (3.70)**	**14.50 (3.74)**	**t = -2.76, p=0.004**
**Dual Task (Cognitive) Cost**			
Velocity Cost (SD)	**-12.11 (0.36)%**	**-24.11 (16.46)%**	**t = 2.29, p=0.01**
Single Support Time Cost (IQR)	6.57 (1.43, 12.53)%	8.45 (1.51, 21.17)%	z = -1.10, p=0.27
Swing Time Cost	8.25 (1.19, 13.66)%	12.12 (3.95, 24.11)%	z -1.82, p = 0.07
Step Time Cost	11.19 (0.93, 19.56)%	16.60 (5.97, 26.58)%	z = -1.65, p=0.10
Stride Time Cost	10.0 (1.78, 18.79)%	16.09 (5.11, 30.17)%	z = -1.70, p=0.09
Cadence Cost	**-9.58 (-14.01, -0.01)%**	**-15.12 (-22.0, -7.15)%**	**z = 2.66, p=0.01**
Stance Time Cost	12.89 (4.34, 22.78)%	18.0% (6.41, 29.55)%	z = -1.41, p=0.16
Stride Length Standard Deviation Cost	28.59 (-56.21, 213.8)%	27.66 (-29.40, 161.5)%	z = -0.41, p=0.68
Step Length Standard Deviation Cost	-11.34 (-45.23, 70.83)%	53.75 (-29.01, 193.5)%	z = -1.73, p=0.08
Stride Velocity Standard Deviation Cost	3.09 (-59.01, 63.78)%	9.03 (-50.01, 109.2)%	z = -0.35, p=0.73
Stride Time Standard Deviation Cost	-4.44 (-44.51, 124.8)%	57.90 (-17.65, 270.6)%	z = -1.99, p=0.04
Step Time Standard Deviation Cost	21.13 (-39.62, 109.1)%	172 (6, 610)%	z = -2.92, p=0.003
Swing Time Standard Deviation Cost	70.00 (-10.77, 183.3)%	77.29 (-15.76, 362.5)%	z = -0.32, p=0.75

Stance Time Standard Deviation Cost	-3.50 (-41.5, 150.0)%	98.7% (-13.80, 294.3)%	z = -1.89, p=0.06
Single Support Time Standard Deviation Cost	38.46 (-15.38, 176.5)%	80.00 (-17.39, 216.67)%	z = -0.94, p = 0.35
Double Support Time Standard Deviation Cost	18.18 (-46.15, 200.0)%	66.92 (-22.48, 301.19)%	z = -1.03, p=0.31
Single Support Time % of Gait Cycle Cost	9.61 (-2.05, 19.62)%	5.73 (-3.54, 20.53)%	z = 0.33, p = 0.74
Swing Time % of Gait Cycle Cost	-2.33 (-5.89, 0.29)%	-3.28 (-7.34, 2.94)%	z = -0.17, p = 0.86
Stance Time % of Gait Cycle Cost	12.89 (4.34, 22.78)%	18.00 (6.41, 29.55)%	z = -1.41, p = 0.16
Double Support Time % of Gait Cycle Cost	18.18 (-46.15, 200)%	66.92 (-22.48, 301.2)%	z = 0.33, p = 0.74
Double Support Time Cost	9.62 (-2.05, 19.63)%	5.73 (-3.54, 20.53)%	z = -1.10, p=0.27
Stride Length Cost	-9.74 (-16.56, -2.39)%	-8.11 (-14.77, -2.62)	z = -0.66, p = 0.51
Step Length Cost	-8.20 (-16.81, -1.85)%	-7.35 (-13.08, 1.35)%	z = -0.51, p=0.61
Stride Width Cost	6.46 (3.22, 19.71)%	5.06 (-8.17, 14.30)%	z = 1.08, p = 0.28
Double Support Gait Cycle Cost	11.06 (-1.23, 17.7)%	7.58 (0.57, 18.81)%	z = 0.46, p = 0.65

Differences in spatiotemporal gait characteristics were assessed in 65 individuals with Type 2 Diabetes Mellitus (T2DM) and 37 Healthy Controls (HC) across three walking bouts (usual pace, maximal pace, cognitive dual-task pace). Results are compared between individuals with T2DM and HC using t-tests and wilcoxon rank-sum tests as appropriate. Results are presented as medians with interquartile ranges for non-parametrically distributed parameters and as arithmetic means with standard deviations for normally distributed variables.

Table S2

"Usual" Pace Walk						
	Paired Associates Learning		Spatial Working Memory		Delayed Pattern Recognition	
	Model 1<br>β (95% CI)	Model 2<br>β (95% CI)	Model 1<br>β (95% CI)	Model 2<br>β (95% CI)	Model 1<br>β (95% CI)	Model 2<br>β (95% CI)
**Velocity**	**0.29 (0.07, 0.45)****	**0.20 (0.01, 0.39)***	0.06 (-0.14, 0.26)	0.09 (-0.11, 0.30)	0.06 (-0.14, 0.26)	0.01 (-0.19, 0.21)
**Single Support Time**	0.15 (-0.05, 0.34)	0.12 (-0.14, 0.38)	-0.17 (-0.37, 0.02)	-0.17 (-0.43, 0.10)	0.17 (-0.02, 0.37)	0.19 (-0.07, 0.46)
**Swing Time**	0.10 (-0.10, 0.30)	0.13 (-0.13, 0.39)	-0.13 (-0.33, 0.06)	-0.16 (-0.43, 0.11)	**0.20 (-0.01, 0.39)***	**0.32 (0.06, 0.58)***
**Step Time**	-0.13 (-0.32, 0.07)	-0.15 (-0.36, 0.05)	**-0.24 (-0.43, -0.04)***	**-0.24 (-0.44, -0.03)***	0.06 (-0.14, 0.25)	0.02 (-0.19, 0.24)
**Stride Time**	0.02 (-0.18, 0.21)	-0.03 (-0.23, 0.17)	**-0.20 (-0.39, -0.01)***	-0.18 (-0.38, 0.03)	0.15 (-0.05, 0.34)	0.12 (-0.08, 0.33)
**Cadence**	0.05 (-0.15, 0.24)	0.12 (-0.10, 0.34)	**0.28 (0.09, 0.47)****	**0.31 (0.09, 0.53)****	-0.16 (-0.36, 0.03)	-0.16 (-0.39, 0.07)
**Stance Time**	0.01 (-0.19, 0.20)	-0.05 (-0.24, 0.15)	-0.18 (-0.38, 0.01)	-0.17 (-0.37, 0.03)	0.13 (-0.07, 0.33)	0.10 (-0.10, 0.30)
**Stride Length SD**	0.03 (-0.17, 0.22)	-0.01 (-0.20, 0.19)	-0.06 (-0.26, 0.13)	-0.05 (-0.25, 0.14)	0.01 (-0.19, 0.21)	0.01 (-0.19, 0.21)
**Step Length SD**	0.04 (-0.16, 0.24)	0.01 (-0.19, 0.20)	-0.06 (-0.26, 0.14)	-0.04 (-0.24, 0.15)	0.07 (-0.13, 0.27)	0.06 (-0.14, 0.26)
**Stride Velocity SD**	0.03 (-0.16, 0.23)	0.00 (-0.19, 0.20)	-0.12 (-0.32, 0.08)	-0.13 (-0.32, 0.07)	0.03 (-0.17, 0.23)	0.02 (-0.18, 0.22)
**Stride Time SD**	-0.02 (-0.22, 0.18)	-0.04 (-0.24, 0.15)	-0.15 (-0.35, 0.04)	-0.17 (-0.36, 0.03)	0.11 (-0.08, 0.31)	0.09 (-0.10, 0.29)
**Step Time SD**	-0.16 (-0.35, 0.04)	-0.15 (-0.34, 0.05)	-0.18 (-0.38, 0.01)	-0.19 (-0.39, 0.01)	0.01 (-0.19, 0.21)	-0.01 (-0.21, 0.19)
**Swing Time SD**	0.03 (-0.17, 0.23)	0.06 (-0.13, 0.25)	-0.07 (-0.27, 0.12)	-0.08 (-0.28, 0.11)	-0.01 (-0.20, 0.19)	0.01 (-0.19, 0.21)
**Stance Time SD**	-0.02 (-0.22, 0.18)	-0.04 (-0.23, 0.15)	-0.15 (-0.35, 0.04)	-0.16 (-0.36, 0.03)	0.11 (-0.08, 0.31)	0.09 (-0.10, 0.29)
**SST SD**	-0.11 (-0.31, 0.09)	-0.09 (-0.28, 0.10)	-0.16 (-0.35, 0.04)	-0.17 (-0.37, 0.02)	-0.10 (-0.30, 0.10)	-0.07 (-0.26, 0.13)
**DST SD**	-0.06 (-0.26, 0.14)	-0.08 (-0.27, 0.11)	-0.17 (-0.36, 0.03)	-0.16 (-0.36, 0.03)	0.14 (-0.06, 0.33)	0.16 (-0.04, 0.36)
**SST % of GC**	0.08 (-0.12, 0.28)	0.08 (-0.13, 0.28)	0.06 (-0.14, 0.26)	0.08 (-0.13, 0.29)	-0.02 (-0.21, 0.18)	-0.03 (-0.24, 0.18)
**Swing Time % GC**	0.03 (-0.17, 0.23)	0.09 (-0.12, 0.30)	0.12 (-0.08, 0.31)	0.10 (-0.11, 0.31)	0.01 (-0.19, 0.21)	0.05 (-0.16, 0.26)
**Stance Time % GC**	-0.03 (-0.23, 0.17)	-0.09 (-0.30, 0.12)	-0.12 (-0.31, 0.08)	-0.10 (-0.31, 0.11)	-0.01 (-0.21, 0.19)	-0.05 (-0.26, 0.16)
**DST % of GC**	-0.09 (-0.29, 0.11)	-0.12 (-0.35, 0.12)	0.04 (-0.16, 0.24)	0.10 (-0.14, 0.34)	-0.16 (-0.36, 0.03)	-0.18 (-0.42, 0.05)
**DST**	-0.02 (-0.22, 0.17)	-0.10 (-0.34, 0.13)	-0.11 (-0.31, 0.08)	-0.05 (-0.29, 0.19)	-0.02 (-0.22, 0.18)	-0.04 (-0.28, 0.20)
**Stride Length**	**0.25 (0.06, 0.45)***	0.16 (-0.06, 0.39)	**-0.25 (-0.45, -0.06)****	**-0.24 (-0.47, -0.01)***	**0.26 (0.06, 0.45)****	0.20 (-0.03, 0.43)
**Step Length**	**0.26 (0.06, 0.45)****	0.18 (-0.04, 0.40)	**-0.23 (-0.42, -0.04)***	-0.21 (-0.43, 0.02)	**0.23 (-0.03, 0.42)***	0.16 (-0.06, 0.39)
**Stride Width**	0.06 (-0.13, 0.26)	0.01 (-0.24, 0.25)	**-0.27 (-0.46, -0.08)****	**-0.32 (-0.56, -0.08)***	0.04 (-0.16, 0.24)	0.08 (-0.17, 0.33)
	Reaction Time Task		Rapid Visual Processing		One-Touch Stockings of Cambridge	
	Model 1<br>β (95% CI)	Model 2<br>β (95% CI)	Model 1<br>β (95% CI)	Model 2<br>β (95% CI)	Model 1<br>β (95% CI)	Model 2<br>β (95% CI)
**Velocity**	-0.17 (-0.37, 0.02)	-0.09 (-0.30, 0.10)	0.15 (-0.04, 0.35)	0.06 (-0.13, 0.25)	0.09 (-0.11, 0.29)	0.04 (-0.16, 0.24)

**Single Support Time**	0.09 (-0.11, 0.29)	0.02 (-0.26, 0.27)	-0.02 (-0.22, 0.18)	0.01 (-0.24, 0.26)	0.12 (-0.08, 0.32)	0.23 (-0.03, 0.49)
**Swing Time**	0.15 (-0.05, 0.35)	0.06 (-0.20, 0.33)	-0.07 (-0.27, 0.13)	0.02 (-0.24, 0.27)	0.07 (-0.13, 0.27)	0.23 (-0.03, 0.49)
**Step Time**	-0.04 (-0.23, 0.16)	-0.13 (-0.34, 0.07)	0.11 (-0.08, 0.31)	0.17 (-0.03, 0.37)	0.03 (-0.18, 0.23)	0.12 (-0.07, 0.32)
**Stride Time**	0.08 (-0.12, 0.28)	0.03 (-0.17, 0.23)	0.03 (-0.17, 0.22)	0.00 (-0.19, 0.20)	0.13 (-0.07, 0.32)	0.13 (-0.08, 0.33)
**Cadence**	-0.07 (-0.27, 0.13)	0.05 (-0.18, 0.28)	-0.06 (-0.26, 0.14)	-0.10 (-0.32, 0.12)	-0.09 (-0.29, 0.11)	-0.11 (-0.34, 0.11)
**Stance Time**	0.06 (-0.14, 0.26)	0.02 (-0.17, 0.22)	0.04 (-0.16, 0.24)	0.00 (-0.06, -0.01)	0.12 (-0.08, 0.32)	0.11 (-0.09, 0.30)
**Stride Length SD**	-0.09 (-0.29, 0.11)	-0.10 (-0.29, 0.10)	0.11 (-0.09, 0.30)	0.07 (-0.12, 0.26)	0.14 (-0.06, 0.33)	0.12 (-0.08, 0.31)
**Step Length SD**	-0.11 (-0.31, 0.08)	-0.12 (-0.32, 0.07)	0.17 (-0.03, 0.36)	0.12 (-0.06, 0.31)	0.18 (-0.01, 0.38)	0.15 (-0.04, 0.35)
**Stride Velocity SD**	-0.06 (-0.26, 0.14)	-0.04 (-0.23, 0.15)	0.11 (-0.09, 0.31)	0.05 (-0.14, 0.23)	0.19 (-0.00, 0.39)	0.17 (-0.02, 0.36)
**Stride Time SD**	-0.02 (-0.22, 0.18)	-0.01 (-0.20, 0.18)	0.08 (-0.12, 0.28)	0.04 (-0.14, 0.23)	0.12 (-0.08, 0.32)	0.12 (-0.07, 0.31)
**Step Time SD**	-0.11 (-0.31, 0.09)	-0.15 (-0.34, 0.05)	0.15 (-0.05, 0.34)	0.18 (-0.01, 0.36)	0.01 (-0.19, 0.21)	0.02 (-0.18, 0.22)
**Swing Time SD**	0.06 (-0.14, 0.25)	0.02 (-0.17, 0.22)	-0.05 (-0.25, 0.14)	0.00 (-0.18, 0.19)	-0.13 (-0.33, 0.07)	-0.10 (-0.29, 0.09)
**Stance Time SD**	-0.02 (-0.22, 0.18)	-0.02 (-0.21, 0.18)	0.08 (-0.11, 0.28)	0.05 (-0.14, 0.23)	0.13 (-0.07, 0.32)	0.13 (-0.07, 0.32)
**SST SD**	-0.09 (-0.29, 0.10)	-0.16 (-0.35, 0.04)	0.05 (-0.15, 0.25)	0.11 (-0.07, 0.30)	0.08 (-0.12, 0.27)	0.12 (-0.07, 0.31)
**DST SD**	-0.10 (-0.30, 0.09)	-0.12 (-0.32, 0.08)	0.05 (-0.15, 0.24)	0.03 (-0.16, 0.22)	**0.27 (0.08, 0.46)****	**0.25 (0.06, 0.44)****
**Swing Time % GC**	-0.07 (-0.27, 0.13)	-0.08 (-0.29, 0.13)	-0.03 (-0.23, 0.17)	0.08 (-0.12, 0.28)	-0.07 (-0.26, 0.13)	0.01 (-0.20, 0.22)
**Stance Time % GC**	0.07 (-0.13, 0.27)	0.08 (-0.13, 0.29)	0.03 (-0.17, 0.23)	-0.08 (-0.06, -0.01)	0.07 (-0.13, 0.27)	-0.01 (-0.22, 0.20)
**DST % of GC**	0.16 (-0.03, 0.36)	0.13 (-0.10, 0.37)	-0.05 (-0.25, 0.14)	-0.08 (-0.31, 0.14)	-0.10 (-0.30, 0.10)	-0.22 (-0.45, 0.01)
**DST**	**0.25 (0.05, 0.43)***	0.20 (-0.04, 0.43)	-0.10 (-0.29, 0.10)	-0.19 (-0.41, 0.04)	-0.00 (-0.20, 0.20)	-0.11 (-0.35, 0.12)
**Stride Length**	-0.17 (-0.36, 0.03)	**-0.24 (-0.47,-0.02)***	**0.28 (-0.09, 0.47)****	**0.24 (-0.03, 0.46)***	**0.22 (0.03, 0.41)***	0.22 (-0.01, 0.44)
**Step Length**	-0.15 (-0.35, 0.04)	-0.21 (-0.43, 0.01)	**0.24 (0.05, 0.43)***	0.20 (-0.01, 0.40)	0.18 (-0.01, 0.38)	0.17 (-0.05, 0.39)
**Stride Width**	0.08 (-0.12, 0.28)	-0.09 (-0.34, 0.16)	0.04 (-0.16, 0.24)	0.03 (-0.21, 0.27)	0.01 (-0.18, 0.21)	-0.08 (-0.33, 0.17)
**"Maximal" Pace Walk**						
	**Paired Associates Learning**		**Spatial Working Memory**		**Delayed Pattern Recognition**	
	**Model 1**<br>**β (95% CI)**	**Model 2**<br>**β (95% CI)**	**Model 1**<br>**β (95% CI)**	**Model 2**<br>**β (95% CI)**	**Model 1**<br>**β (95% CI)**	**Model 2**<br>**β (95% CI)**
**Velocity**	**0.21 (0.02, 0.40)***	0.08 (-0.12, 0.29)	**-0.22 (-0.41, -0.02)***	-0.17 (-0.38, 0.04)	0.05 (-0.14, 0.25)	-0.08 (-0.29, 0.13)
**Single Support Time**	0.15 (-0.05, 0.34)	0.15 (-0.07, 0.36)	-0.04 (-0.25, 0.15)	-0.02 (0.24, 0.20)	**0.27 (0.08, 0.46)****	**0.32 (0.11, 0.53)****
**Swing Time**	0.02 (-0.18, 0.21)	-0.02 (-0.25, 0.22)	-0.11 (-0.31, 0.09)	-0.12 (-0.36, 0.12)	**0.22 (0.02, 0.41)***	**0.30 (0.07, 0.53)***
**Step Time**	-0.03 (-0.23, 0.17)	-0.03 (-0.23, 0.18)	0.04 (-0.16, 0.24)	0.06 (-0.14, 0.27)	**0.20 (0.01, 0.39)***	**0.24 (0.04, 0.45)**
**Stride Time**	0.10 (-0.10, 0.29)	0.07 (-0.12, 0.27)	-0.09 (-0.28, 0.11)	-0.05 (-0.26, 0.16)	**0.21 (0.01, 0.40)***	**0.20 (0.00, 0.40)***
**Cadence**	-0.07 (-0.27, 0.12)	-0.06 (-0.27, 0.14)	0.06 (-0.14, 0.26)	0.02 (-0.19, 0.23)	**-0.27 (-0.46, -0.08)****	**-0.30 (-0.50, -0.10)****

**Stance Time**	0.11 (-0.08, 0.31)	0.09 (-0.10, 0.28)	-0.06 (-0.26, 0.13)	-0.02 (-0.22, 0.17)	0.17 (-0.02, 0.37)	0.16 (-0.04, 0.35)
**Stride Length SD**	0.03 (-0.17, 0.22)	0.03 (-0.16, 0.23)	0.00 (-0.20, 0.20)	-0.00 (-0.20, 0.20)	-0.01 (-0.20, 0.19)	0.03 (-0.17, 0.23)
**Step Length SD**	0.05 (-0.15, 0.25)	0.03 (-0.17, 0.23)	-0.10 (-0.30, 0.10)	-0.09 (-0.29, 0.11)	0.04 (-0.16, 0.24)	0.07 (0.13, 0.28)
**Stride Velocity SD**	0.17 (-0.03, 0.36)	0.12 (-0.07, 0.31)	-0.08 (-0.27, 0.13)	-0.05 (-0.25, 0.15)	0.06 (-0.14, 0.26)	0.03 (-0.17, 0.23)
**Stride Time SD**	0.18 (-0.02, 0.37)	0.14 (-0.05, 0.33)	-0.09 (-0.29, 0.11)	-0.06 (-0.26, 0.14)	0.09 (-0.10, 0.29)	0.05 (-0.15, 0.24)
**Step Time SD**	-0.02 (-0.22, 0.18)	-0.01 (-0.20, 0.18)	0.07 (-0.12, 0.27)	0.08 (-0.12, 0.27)	0.12 (-0.08, 0.32)	0.13 (-0.06, 0.33)
**Swing Time SD**	**-0.22 (-0.41, -0.03)***	-0.16 (-0.36, 0.03)	-0.13 (-0.33, 0.07)	-0.17 (-0.37, 0.02)	-0.13 (-0.33, 0.06)	-0.07 (-0.27, 0.13)
**Stance Time SD**	0.18 (-0.02, 0.37)	0.14 (-0.06, 0.33)	-0.10 (-0.30, 0.10)	-0.07 (-0.27, 0.13)	0.10 (-0.09, 0.30)	0.05 (-0.15, 0.25)
**SST SD**	0.09 (-0.11, 0.29)	0.13 (-0.06, 0.32)	-0.07 (-0.27, 0.13)	-0.08 (-0.28, 0.11)	-0.01 (-0.20, 0.19)	0.02 (-0.17, 0.22)
**DST SD**	-0.16 (-0.36, 0.03)	-0.13 (-0.32, 0.07)	0.04 (-0.16, 0.24)	0.05 (-0.15, 0.25)	-0.15 (-0.35, 0.05)	-0.11 (-0.31, 0.09)
**SST % of GC**	0.11 (-0.09, 0.31)	0.11 (-0.09, 0.30)	0.05 (-0.15, 0.25)	0.04 (-0.16, 0.25)	0.10 (-0.11, 0.29)	0.92 (-0.05, 0.01)
**Swing Time % of GC**	-0.09 (-0.29, 0.11)	-0.11 (-0.32, 0.10)	-0.04 (-0.24, 0.16)	-0.07 (-0.29, 0.14)	-0.00 (-0.20, 0.20)	-0.01 (-0.22, 0.21)
**Stance Time % of GC**	0.09 (-0.11, 0.29)	0.11 (-0.10, 0.32)	0.04 (-0.16, 0.24)	0.08 (-0.14, 0.29)	0.01 (-0.19, 0.20)	0.01 (-0.20, 0.22)
**DST % of GC**	-0.18 (-0.38, 0.01)	-0.18 (-0.41, 0.07)	0.03 (-0.17, 0.23)	0.01 (-0.24, 0.26)	**-0.22 (-0.42, -0.03)**	-0.23 (-0.47, 0.02)
**DST**	-0.16 (-0.35, 0.04)	-0.13 (-0.34, 0.09)	0.07 (-0.13, 0.26)	0.08 (-0.14, 0.30)	-0.09 (-0.29, 0.11)	-0.02 (-0.25, 0.20)
**Stride Length**	**0.33 (0.14, 0.52)****	**0.26 (0.01, 0.52)***	**-0.31 (-0.50, -0.13)****	**-0.35 (-0.60, -0.09)****	**0.27 (0.08, 0.46)****	0.23 (-0.03, 0.49)
**Step Length**	**0.32 (0.13, 0.51)****	0.24 (-0.01, 0.49)	**-0.30 (-0.48, -0.10)****	**-0.30 (-0.56, -0.05)***	**0.27 (0.08, 0.46)****	0.21 (-0.04, 0.47)
**Stride Width**	0.10 (-0.09, 0.30)	0.03 (-0.21, 0.26)	**-0.22 (-0.42, -0.03)***	**-0.23 (-0.47, -0.00)***	0.03 (-0.16, 0.23)	0.03 (-0.20, 0.27)
	**Reaction Time Task**		**Rapid Visual Processing**		**One-Touch Stockings of Cambridge**	
	**Model 1**<br>**β (95% CI)**	**Model 2**<br>**β (95% CI)**	**Model 1**<br>**β (95% CI)**	**Model 2**<br>**β (95% CI)**	**Model 1**<br>**β (95% CI)**	**Model 2**<br>**β (95% CI)**
**Velocity**	0.05 (-0.14, 0.25)	-0.08 (-0.29, 0.13)	**0.23 (0.04, 0.42)***	0.09 (-0.11, 0.29)	0.11 (-0.08, 0.31)	0.01 (-0.20, 0.22)
**Single Support Time**	**0.27 (0.08, 0.46)****	**0.32 (0.11, 0.53)****	0.04 (-0.16, 0.24)	0.11 (-0.10, 0.31)	0.08 (-0.11, 0.28)	0.15 (-0.07, 0.36)
**Swing Time**	**0.22 (0.02, 0.41)***	**0.30 (0.07, 0.53)****	-0.06 (-0.26, 0.14)	0.02 (-0.24, 0.27)	0.06 (-0.14, 0.25)	0.16 (-0.07, 0.39)
**Step Time**	**0.20 (0.00, 0.39)***	**0.24 (0.04, 0.45)***	-0.00 (-0.20, 0.20)	0.07 (-0.12, 0.27)	0.11 (-0.09, 0.31)	0.17 (-0.03, 0.37)
**Stride Time**	**0.21 (0.01, 0.40)***	**0.20 (0.00, 0.40)***	0.01 (-0.19, 0.21)	0.03 (-0.16, 0.23)	0.09 (-0.11, 0.28)	0.10 (-0.10, 0.29)
**Cadence**	**-0.27 (-0.46, -0.08)****	**-0.30 (-0.50, -0.10)****	-0.03 (-0.22, 0.18)	-0.08 (-0.28, 0.12)	-0.13 (-0.32, 0.07)	-0.16 (-0.37, 0.04)
**Stance Time**	0.17 (-0.02, 0.37)	0.16 (-0.04, 0.35)	0.04 (-0.16, 0.23)	0.04 (-0.15, 0.22)	0.09 (-0.11, 0.29)	0.07 (-0.12, 0.27)
**Stride Length SD**	-0.01 (-0.20, 0.19)	0.03 (-0.17, 0.23)	0.06 (-0.13, 0.26)	0.13 (-0.06, 0.32)	-0.04 (-0.24, 0.15)	-0.01 (-0.21, 0.19)
**Step Length SD**	0.04 (-0.16, 0.24)	0.07 (-0.13, 0.28)	0.03 (-0.17, 0.23)	0.09 (-0.11, 0.28)	-0.07 (-0.26, 0.13)	-0.06 (-0.26, 0.15)
**Stride Velocity SD**	0.06 (-0.14, 0.26)	0.03 (-0.17, 0.23)	**0.23 (-0.04, 0.42)***	**0.18 (0.00, 0.36)***	0.09 (-0.12, 0.28)	0.05 (-0.15, 0.24)
**Stride Time SD**	0.09 (-0.10, 0.29)	0.05 (-0.15, 0.25)	0.15 (-0.04, 0.35)	0.10 (-0.08, 0.29)	0.11 (-0.09, 0.30)	0.06 (-0.14, 0.26)

**Step Time SD**	0.12 (-0.08, 0.32)	0.14 (-0.06, 0.33)	0.07 (-0.13, 0.26)	0.10 (-0.08, 0.28)	0.12 (-0.08, 0.32)	0.14 (-0.05, 0.33)
**Swing Time SD**	-0.13 (-0.33, 0.06)	-0.07 (-0.27, 0.13)	-0.13 (-0.33, 0.07)	-0.03 (-0.22, 0.16)	-0.17 (-0.36, 0.03)	-0.11 (-0.31, 0.09)
**Stance Time SD**	0.10 (-0.09, 0.30)	0.05 (-0.15, 0.25)	0.16 (-0.03, 0.36)	0.11 (-0.08, 0.29)	0.11 (-0.09, 0.31)	0.06 (-0.14, 0.26)
**SST SD**	-0.01 (-0.20, 0.19)	0.02 (-0.17, 0.22)	0.10 (-0.10, 0.30)	0.14 (-0.05, 0.32)	-0.07 (-0.27, 0.12)	-0.06 (-0.26, 0.13)
**DST SD**	-0.15 (-0.35, 0.05)	-0.11 (-0.31, 0.09)	-0.04 (-0.24, 0.16)	0.05 (-0.15, 0.23)	-0.13 (-0.32, 0.07)	-0.11 (-0.31, 0.09)
**SST % of GC**	0.10 (-0.11, 0.30)	0.09 (-0.11, 0.29)	0.08 (-0.12, 0.28)	0.10 (-0.09, 0.29)	0.02 (-0.18, 0.22)	0.05 (-0.15, 0.25)
**Swing Time % of GC**	-0.00 (-0.20, 0.20)	-0.01 (-0.22, 0.21)	-0.06 (-0.25, 0.14)	-0.02 (-0.22, 0.18)	-0.03 (-0.23, 0.16)	0.03 (-0.18, 0.24)
**Stance Time % of GC**	0.00 (-0.19, 0.20)	-0.01 (-0.20, 0.22)	0.05 (-0.14, 0.25)	0.02 (-0.06, -0.01)	0.04 (-0.16, 0.24)	-0.03 (-0.24, 0.18)
**DST % of GC**	**-0.22 (-0.42, -0.03)***	-0.23 (-0.47, 0.02)	-0.17 (-0.37, 0.03)	-0.20 (-0.43, 0.04)	-0.19 (-0.38, 0.01)	-0.31 (-0.54, -0.07)
**DST**	-0.09 (-0.29, 0.11)	-0.03 (-0.25, 0.20)	-0.16 (-0.36, 0.03)	-0.12 (-0.33, 0.08)	-0.12 (-0.31, 0.08)	-0.14 (-0.35, 0.08)
**Stride Length**	**0.27 (0.08, 0.47)****	0.23 (-0.03, 0.49)	**0.29 (0.10, 0.48)****	**0.25 (0.10, 0.50)***	**0.24 (0.05, 0.43)***	0.24 (-0.02, 0.50)
**Step Length**	**0.27 (-0.08, 0.46)****	0.21 (-0.04, 0.47)	**0.29 (0.09, 0.47)****	0.22 (-0.02, 0.46)	**0.22 (0.03, 0.42)***	0.20 (-0.05, 0.45)
**Stride Width**	0.03 (-0.16, 0.23)	0.03 (-0.20, 0.27)	0.14 (-0.05, 0.34)	0.13 (-0.09, 0.35)	0.03 (-0.17, 0.23)	-0.04 (-0.27, 0.19)

The relationships between spatiotemporal gait parameters on usual and maximal pace walking and cognitive function were assessed in 102 individuals (with and without T2DM). Linear regression was used with gait characteristic as the predictor (independent) variable and cognitive performance as the outcome (dependent variable). Results are presented as Beta Coefficients (β) with appropriate 95% Confidence Interval (95% CI). Model 1 is unadjusted whilst Model 2 adjusted for age, sex, BMI, T2DM status and education ***p<0.05, **p<0.01, ***p<0.001.**

Cognitive Dual Task Walk						
	Paired Associates Learning		Spatial Working Memory		Delayed Pattern Recognition	
	Model 1<br>β (95% CI)	Model 2<br>β (95% CI)	Model 1<br>β (95% CI)	Model 2<br>β (95% CI)	Model 1<br>β (95% CI)	Model 2<br>β (95% CI)
**Velocity**	0.11 (-0.09, 0.31)	0.10 (-0.10, 0.30)	-0.04 (-0.24, 0.15)	-0.07 (-0.27, 0.13)	0.18 (-0.01, 0.38)	0.17 (-0.03, 0.37)
**Single Support Time**	0.02 (-0.18, 0.22)	-0.02 (-0.22, 0.18)	-0.11 (-0.31, 0.09)	-0.07 (-0.27, 0.13)	0.01 (-0.19, 0.20)	-0.03 (-0.23, 0.18)
**Swing Time**	0.08 (-0.12, 0.28)	0.02 (-0.18, 0.21)	-0.09 (-0.29, 0.11)	-0.03 (-0.24, 0.17)	-0.03 (-0.22, 0.17)	-0.09 (-0.29, 0.11)
**Step Time**	0.03 (-0.16, 0.23)	-0.01 (-0.20, 0.19)	-0.09 (-0.28, 0.10)	-0.05 (-0.25, 0.15)	-0.08 (-0.27, 0.12)	-0.11 (-0.31, 0.08)
**Stride Time**	-0.06 (-0.25, 0.14)	-0.12 (-0.31, 0.08)	-0.01 (-0.21, 0.19)	0.05 (-0.15, 0.25)	-0.15 (-0.34, 0.05)	**-0.20 (-0.40, -0.01)***
**Cadence**	-0.02 (-0.22, 0.18)	0.03 (-0.17, 0.23)	0.07 (-0.13, 0.27)	-0.00 (-0.21, 0.20)	0.02 (-0.19, 0.21)	0.05 (-0.16, 0.25)
**Stance Time**	-0.12 (-0.31, 0.08)	-0.16 (-0.35, 0.03)	0.04 (-0.16, 0.24)	0.08 (-0.11, 0.28)	-0.20 (-0.39, 0.00)	**-0.23 (-0.42, -0.04)***
**Stride Length SD**	-0.02 (-0.22, 0.18)	-0.06 (-0.25, 0.13)	0.04 (-0.16, 0.24)	0.05 (-0.15, 0.25)	-0.14 (-0.33, 0.06)	-0.15 (-0.34, 0.05)
**Step Length SD**	-0.01 (-0.21, 0.19)	-0.03 (-0.22, 0.17)	-0.01 (-0.20, 0.19)	-0.01 (-0.21, 0.19)	-0.05 (-0.25, 0.15)	-0.04 (-0.24, 0.16)
**Stride Velocity SD**	-0.03 (-0.23, 0.16)	-0.06 (-0.25, 0.13)	0.04 (-0.16, 0.24)	0.06 (-0.14, 0.25)	-0.16 (-0.35, 0.04)	-0.18 (-0.37, 0.02)
**Stride Time SD**	0.01 (-0.18, 0.21)	-0.02 (-0.22, 0.17)	0.00 (-0.20, 0.20)	0.04 (-0.16, 0.24)	-0.14 (-0.33, 0.06)	-0.17 (-0.36, 0.01)
**Step Time SD**	0.15 (-0.04, 0.35)	0.14 (-0.05, 0.32)	-0.15 (-0.34, 0.05)	-0.12 (-0.32, 0.07)	0.02 (-0.18, 0.21)	-0.00 (-0.20, 0.19)
**Swing Time SD**	0.10 (-0.09, 0.30)	0.04 (-0.15, 0.23)	-0.06 (-0.26, 0.14)	-0.02 (-0.22, 0.18)	-0.08 (-0.28, 0.11)	-0.15 (-0.35, 0.05)
**Stance Time SD**	-0.15 (-0.35, 0.05)	-0.14 (-0.33, 0.05)	0.13 (-0.07, 0.33)	0.15 (-0.05, 0.34)	**-0.20 (-0.40, -0.01)***	**-0.20 (-0.39, -0.01)***
**SST SD**	-0.02 (-0.22, 0.18)	-0.02 (-0.21, 0.17)	-0.05 (-0.25, 0.15)	-0.05 (-0.25, 0.14)	-0.09 (-0.29, 0.11)	-0.08 (-0.28, 0.11)
**DST SD**	-0.05 (-0.25, 0.14)	-0.05 (-0.24, 0.14)	-0.03 (-0.22, 0.17)	-0.02 (-0.21, 0.18)	-0.13 (-0.33, 0.07)	-0.13 (-0.32, 0.07)
**SST % of GC**	0.14 (-0.06, 0.34)	0.15 (-0.04, 0.34)	-0.15 (-0.35, 0.04)	-0.16 (-0.35, 0.04)	**0.22 (0.03, 0.41)***	**0.23 (0.04, 0.42)***
**Swing Time % GC**	**0.21 (0.02, 0.40)***	**0.01 (0.05, 0.43)***	-0.10 (-0.30, 0.10)	-0.12 (-0.32, 0.08)	0.18 (-0.01, 0.38)	**0.20 (0.01, 0.41)***
**Stance Time % GC**	**-0.21 (-0.40, -0.02)***	**-0.24 (-0.43, -0.05)***	0.10 (-0.10, 0.30)	0.12 (-0.08, 0.32)	-0.18 (-0.38, 0.01)	**-0.20 (-0.40, -0.01)***
**DST % of GC**	-0.02 (-0.22, 0.18)	-0.02 (-0.21, 0.17)	-0.13 (-0.33, 0.06)	-0.12 (-0.32, 0.75)	-0.05 (-0.25, 0.15)	-0.04 (-0.24, 0.16)
**DST**	-0.12 (-0.32, 0.08)	-0.15 (-0.34, 0.04)	0.01 (-0.18, 0.21)	0.05 (-0.15, 0.24)	**-0.21 (-0.41, -0.02)***	**-0.24 (-0.43, -0.05)***
**Stride Length**	**0.22 (0.02, 0.41)***	0.16 (-0.04, 0.36)	-0.19 (-0.38, 0.01)	-0.15 (-0.36, 0.06)	**0.28 (0.09, 0.47)****	**0.26 (0.05, 0.46)***
**Step Length**	**0.20 (0.00, 0.39)***	0.15 (-0.06, 0.34)	-0.17 (-0.37, 0.02)	-0.14 (-0.35, 0.06)	**0.23 (0.04, 0.43)***	0.20 (-0.01, 0.41)
**Stride Width**	0.04 (-0.16, 0.24)	-0.06 (-0.28, 0.17)	-0.15 (-0.34, 0.05)	-0.11 (-0.34, 0.12)	-0.03 (-0.22, 0.18)	-0.06 (-0.29, 0.17)
	Reaction Time Task		Rapid Visual Processing		One-Touch Stockings of Cambridge	
	Model 1<br>β (95% CI)	Model 2<br>β (95% CI)	Model 1<br>β (95% CI)	Model 2<br>β (95% CI)	Model 1<br>β (95% CI)	Model 2<br>β (95% CI)
**Velocity**	-0.17 (-0.37, 0.02)	-0.15 (-0.35, 0.04)	**0.23 (0.04, 0.42)***	**0.19 (0.01, 0.37)***	**0.20 (0.00, 0.39)***	**0.21 (0.02, 0.41)***

**Single Support Time**	0.03 (-0.17, 0.23)	-0.01 (-0.21, 0.19)	-0.05 (-0.25, 0.14)	-0.04 (-0.23, 0.15)	0.02 (-0.18, 0.21)	0.01 (-0.20, 0.21)
**Swing Time**	0.01 (-0.18, 0.21)	0.00 (-0.20, 0.20)	-0.12 (-0.32, 0.08)	-0.16 (-0.34, 0.03)	-0.00 (-0.20, 0.19)	-0.04 (-0.24, 0.16)
**Step Time**	-0.01 (-0.21, 0.19)	-0.01 (-0.20, 0.19)	-0.05 (-0.25, 0.15)	-0.07 (-0.25, 0.12)	-0.06 (-0.25, 0.14)	-0.09 (-0.28, 0.11)
**Stride Time**	0.06 (-0.13, 0.26)	0.06 (-0.13, 0.26)	-0.15 (-0.35, 0.05)	**-0.19 (-0.37, -0.01)***	-0.05 (-0.25, 0.15)	-0.09 (-0.29, 0.11)
**Cadence**	-0.07 (-0.26, 0.13)	-0.01 (-0.21, 0.19)	0.07 (-0.13, 0.27)	0.06 (-0.13, 0.25)	0.04 (-0.16, 0.24)	0.07 (-0.13, 0.28)
**Stance Time**	0.07 (-0.13, 0.27)	0.07 (-0.12, 0.27)	-0.14 (-0.34, 0.06)	-0.17, (-0.35, 0.01)	-0.08 (-0.28, 0.12)	-0.12 (-0.31, 0.07)
**Stride Length SD**	-0.13 (-0.32, 0.07)	-0.12 (-0.31, 0.08)	-0.08 (-0.28, 0.11)	-0.12 (-0.31, 0.06)	-0.12 (-0.32, 0.08)	-0.15 (-0.35, 0.04)
**Step Length SD**	-0.17 (-0.36, 0.03)	-0.17 (-0.36, 0.02)	-0.08 (-0.28, 0.11)	-0.09 (-0.27, 0.10)	-0.09 (-0.29, 0.11)	-0.09 (-0.29, 0.10)
**Stride Velocity SD**	-0.10 (-0.30, 0.10)	-0.06 (-0.25, 0.13)	-0.05 (-0.25, 0.15)	-0.09 (-0.28, 0.10)	-0.08 (-0.28, 0.12)	-0.12 (-0.31, 0.07)
**Stride Time SD**	0.09 (-0.10, 0.30)	0.10 (-0.09, 0.30)	-0.19 (-0.39, 0.00)	**-0.22 (-0.40, -0.04)***	-0.12 (-0.32, 0.07)	-0.16 (-0.35, 0.03)
**Step Time SD**	-0.06 (-0.26, 0.13)	-0.05 (-0.24, 0.15)	0.05 (-0.15, 0.25)	0.04 (-0.15, 0.22)	-0.05 (-0.25, 0.14)	-0.08 (-0.27, 0.11)
**Swing Time SD**	-0.04 (-0.24, 0.16)	-0.00 (-0.20, 0.20)	-0.11 (-0.31, 0.09)	**-0.19 (-0.37, -0.01)***	-0.03 (-0.23, 0.17)	-0.09 (-0.28, 0.11)
**Stance Time SD**	**0.27 (0.07, 0.46)****	**0.25 (0.06, 0.43)***	**-0.23 (-0.42, -0.03)***	**-0.18 (-0.36, -0.00)***	-0.19 (-0.38, 0.01)	-0.18 (-0.37, 0.01)
**SST SD**	0.05 (-0.15, 0.24)	0.03 (-0.16, 0.22)	-0.03 (-0.23, 0.16)	-0.03 (-0.20, 0.16)	-0.01 (-0.21, 0.19)	0.01 (-0.19, 0.20)
**DST SD**	-0.00 (-0.20, 0.20)	-0.02 (-0.22, 0.17)	-0.11 (-0.30, 0.09)	-0.08 (-0.26, 0.11)	-0.09 (-0.29, 0.11)	-0.08 (-0.27, 0.12)
**SST % of GC**	-0.02 (-0.22, 0.18)	-0.06 (-0.25, 0.13)	0.12 (-0.07, 0.32)	0.16 (-0.02, 0.35)	0.11 (-0.09, 0.30)	0.14 (-0.06, 0.33)
**Swing Time % GC**	-0.16 (-0.36, 0.04)	-0.19 (-0.38, 0.01)	0.05 (-0.15, 0.25)	0.11 (-0.08, 0.29)	0.09 (-0.10, 0.29)	0.15 (-0.05, 0.35)
**Stance Time % GC**	0.16 (-0.04, 0.36)	0.19 (-0.01, 0.38)	-0.05 (-0.25, 0.15)	-0.11 (-0.30, 0.08)	-0.09 (-0.29, 0.11)	-0.15 (-0.35, 0.05)
**DST % of GC**	0.12 (-0.08, 0.31)	0.09 (-0.10, 0.29)	-0.07 (-0.27, 0.12)	-0.69 (-0.25, 0.12)	-0.01 (-0.21, 0.19)	-0.02 (-0.22, 0.17)
**DST**	0.03 (-0.17, 0.23)	0.04 (-0.16, 0.23)	-0.13 (-0.33, 0.06)	-0.16 (-0.34, 0.02)	-0.09 (-0.29, 0.11)	-0.12 (-0.31, 0.07)
**Stride Length**	-0.19 (-0.39, 0.00)	**-0.28 (-0.48, -0.08)****	**0.30 (0.11, 0.49)****	**0.31 (0.12, 0.50)****	**0.31 (0.13, 0.50)****	**0.34 (0.14, 0.54)****
**Step Length**	-0.23 (-0.42, -0.04)	**-0.30 (-0.50, -0.10)****	**0.31 (0.12, 0.50)****	**0.31 (0.12, 0.49)****	**0.30 (0.11, 0.39)****	**0.31 (0.12, 0.51)****
**Stride Width**	0.19 (-0.01, 0.38)	0.13 (-0.10, 0.35)	-0.04 (-0.24, 0.16)	-0.13 (-0.35, 0.08)	-0.07 (-0.27, 0.12)	-0.19 (-0.42, 0.03)
**Dual Task Cost**						
	**Paired Associates Learning**		**Spatial Working Memory**		**Delayed Pattern Recognition**	
	**Model 1**<br>**β (95% CI)**	**Model 2**<br>**β (95% CI)**	**Model 1**<br>**β (95% CI)**	**Model 2**<br>**β (95% CI)**	**Model 1**<br>**β (95% CI)**	**Model 2**<br>**β (95% CI)**
**Velocity**	-0.07 (-0.27, 0.13)	-0.06 (-0.26, 0.14)	-0.15 (-0.34, 0.05)	-0.19 (-0.39, 0.01)	0.14 (-0.06, 0.34)	-0.02 (-0.05, 0.00)
**Single Support Time**	-0.02 (-0.22, 0.18)	-0.04 (-0.23, 0.15)	-0.06 (-0.26, 0.14)	-0.03 (-0.23, 0.16)	-0.04 (-0.24, 0.15)	-0.06 (-0.25, 0.14)
**Swing Time**	0.05 (-0.15, 0.25)	-0.01 (-0.20, 0.19)	-0.05 (-0.25, 0.15)	-0.00 (-0.20, 0.20)	-0.09 (-0.29, 0.10)	-0.14 (-0.33, 0.06)
**Step Time**	0.04 (-0.16, 0.24)	0.02 (-0.18, 0.21)	-0.02 (-0.21, 0.18)	0.00 (-0.20, 0.20)	-0.13 (-0.32, 0.07)	-0.15 (-0.34, 0.05)
**Stride Time**	-0.09 (-0.29, 0.11)	-0.13 (-0.32, 0.06)	0.07 (-0.13, 0.27)	0.11 (-0.09, 0.30)	**-0.21 (-0.40, -0.02)***	**-0.25 (-0.44, -0.06)***

**Cadence**	-0.09 (-0.28, 0.11)	-0.09 (-0.28, 0.12)	-0.17 (-0.37, 0.02)	**-0.22 (-0.42, -0.02)***	0.12 (-0.08, 0.32)	0.11 (-0.09, 0.32)
**Stance Time**	-0.14 (-0.34, 0.06)	-0.17 (-0.36, 0.02)	0.11 (-0.09, 0.30)	0.13 (-0.07, 0.32)	**-0.24 (-0.43, -0.05)***	**-0.26 (-0.45, -0.07)****
**Stride Length SD**	0.18 (-0.01, 0.38)	0.18 (-0.01, 0.36)	0.14 (-0.05, 0.34)	0.15 (-0.05, 0.34)	0.05 (-0.15, 0.25)	0.05 (-0.14, 0.25)
**Step Length SD**	-0.09 (-0.29, 0.11)	-0.14 (-0.33, 0.05)	0.05 (-0.14, 0.25)	0.07 (-0.13, 0.26)	-0.19 (-0.38, 0.01)	**-0.21 (-0.40, -0.01)***
**Stride Velocity SD**	-0.07 (-0.27, 0.13)	-0.04 (-0.23, 0.15)	-0.04 (-0.24, 0.16)	-0.06 (-0.26, 0.13)	0.05 (-0.15, 0.25)	0.07 (-0.13, 0.26)
**Stride Time SD**	0.10 (-0.10, 0.31)	0.03 (-0.17, 0.23)	-0.11 (-0.31, 0.10)	-0.01 (-0.22, 0.20)	-0.04 (-0.24, 0.16)	-0.12 (-0.32, 0.09)
**Step Time SD**	-0.07 (-0.27, 0.13)	-0.10 (-0.30, 0.10)	0.07 (-0.12, 0.26)	0.07 (-0.12, 0.27)	**-0.21 (-0.40, -0.01)***	**-0.24 (-0.44, -0.06)***
**Swing Time SD**	0.09 (-0.11, 0.30)	0.02 (-0.18, 0.22)	-0.01 (-0.22, 0.20)	0.04 (-0.16, 0.24)	-0.05 (-0.26, 0.16)	-0.10 (-0.31, 0.10)
**Stance Time SD**	0.00 (-0.20, 0.20)	-0.05 (-0.25, 0.15)	-0.05 (-0.25, 0.14)	0.02 (-0.18, 0.22)	-0.13 (-0.33, 0.07)	0.03 (-0.17, 0.24)
**SST SD**	0.05 (-0.16, 0.10)	0.05 (-0.15, 0.25)	-0.10 (-0.30, 0.10)	0.04 (-0.17, 0.24)	0.05 (-0.15, 0.25)	**-0.21 (-0.41, -0.01)***
**DST SD**	0.05 (-0.16, 0.25)	0.05 (-0.15, 0.25)	0.04 (-0.17, 0.24)	0.02 (-0.18, 0.22)	**-0.21 (-0.40, -0.01)***	**-0.20 (-0.40, -0.01)***
**SST % of GC**	-0.19 (-0.39, 0.01)	-0.18 (-0.38, 0.01)	**-0.20 (-0.39, -0.01)***	**-0.22 (-0.41, -0.02)***	**0.22 (-0.02, 0.41)***	**0.22 (0.03, 0.42)***
**Swing Time % of GC**	0.06 (-0.13, 0.26)	0.07 (-0.13, 0.26)	-0.18 (-0.38, 0.02)	-0.18 (-0.37, 0.01)	0.16 (-0.04, 0.35)	0.14 (-0.05, 0.33)
**Stance Time % of GC**	0.09 (-0.11, 0.29)	0.07 (-0.12, 0.26)	0.15 (-0.04, 0.35)	0.14 (-0.05, 0.34)	-0.15 (-0.35, 0.04)	-0.14 (-0.33, 0.05)
**DST % of GC**	-0.18 (-0.38, 0.01)	-0.17 (-0.36, 0.02)	-0.19 (-0.38, 0.01)	-0.19 (-0.38, 0.00)	0.06 (-0.14, 0.26)	0.05 (-0.15, 0.24)
**DST**	-0.11 (-0.31, 0.08)	-0.13 (-0.33, 0.06)	0.06 (-0.14, 0.26)	0.07 (-0.13, 0.26)	**-0.22 (-0.41, -0.02)***	**-0.24 (-0.43, -0.05)***
**Stride Length**	0.05 (-0.15, 0.25)	0.07 (-0.12, 0.26)	-0.01 (-0.21, 0.18)	-0.03 (-0.22, 0.17)	0.11 (-0.08, 0.31)	0.14 (-0.05, 0.33)
**Step Length**	0.02 (-0.18, 0.22)	0.04 (-0.15, 0.23)	-0.01 (-0.21, 0.19)	-0.02 (-0.22, 0.17)	0.06 (-0.14, 0.26)	0.09 (-0.11, 0.28)
**Stride Width**	-0.06 (-0.26, 0.13)	-0.11 (-0.30, 0.09)	0.16 (-0.04, 0.36)	0.16 (-0.04, 0.36)	-0.08 (-0.28, 0.11)	-0.12 (-0.32, 0.08)
	**Reaction Time Task**		**Rapid Visual Processing**		**One-Touch Stockings of Cambridge**	
	**Model 1** β **(95% CI)**	**Model 2** β **(95% CI)**	**Model 1** β **(95% CI)**	**Model 2** β **(95% CI)**	**Model 1** β **(95% CI)**	**Model 2** β **(95% CI)**
**Velocity**	-0.11 (-0.31, 0.09)	-0.13 (-0.33, 0.07)	0.18 (-0.02, 0.38)	0.19 (-0.00, 0.37)	0.18 (-0.02, 0.37)	**0.22 (0.02, 0.41)***
**Single Support Time**	0.00 (-0.20, 0.20)	-0.01 (-0.21, 0.18)	-0.05 (-0.25, 0.14)	-0.05 (-0.23, 0.13)	-0.02 (-0.22, 0.18)	-0.03 (-0.22, 0.16)
**Swing Time**	-0.04 (-0.24, 0.16)	-0.02 (-0.22, 0.17)	-0.11 (-0.31, 0.09)	-0.16 (-0.34, 0.02)	-0.03 (-0.22, 0.17)	-0.08 (-0.27, 0.12)
**Step Time**	-0.05 (-0.25, 0.15)	-0.00 (-0.20, 0.20)	-0.05 (-0.25, 0.15)	-0.08 (-0.26, 0.11)	-0.06 (-0.26, 0.14)	-0.09 (-0.28, 0.11)
**Stride Time**	0.00 (-0.20, 0.20)	0.03 (-0.16, 0.23)	-0.15 (-0.34, 0.05)	-0.18 (-0.36, 0.01)	-0.08 (-0.28, 0.12)	-0.12 (-0.31, 0.08)
**Cadence**	-0.07 (-0.27, 0.13)	-0.08 (-0.29, 0.12)	0.15 (-0.05, 0.35)	0.15 (-0.04, 0.34)	0.12 (-0.08, 0.32)	0.15 (-0.05, 0.35)
**Stance Time**	0.00 (-0.20, 0.20)	0.04 (-0.16, 0.23)	-0.12 (-0.32, 0.07)	-0.14 (-0.33, 0.04)	-0.11 (-0.30, 0.09)	-0.13 (-0.32, 0.06)
**Stride Length SD**	0.15 (-0.05, 0.35)	0.13 (-0.06, 0.32)	0.05 (-0.15, 0.25)	0.05 (-0.13, 0.23)	-0.04 (-0.24, 0.16)	-0.04 (-0.23, 0.16)
**Step Length SD**	-0.13 (-0.33, 0.07)	-0.11 (-0.30, 0.09)	-0.16 (-0.36, 0.04)	**-0.22 (-0.40, -0.03)***	-0.07 (-0.27, 0.12)	-0.10 (-0.29, 0.10)
**Stride Velocity SD**	-0.18 (-0.38, 0.02)	**-0.21 (-0.39, -0.20)***	0.09 (-0.11, 0.29)	0.12 (-0.06, 0.31)	-0.02 (-0.22, 0.18)	0.01 (-0.18, 0.21)

**Stride Time SD**	-0.10 (-0.31, 0.10)	-0.13 (-0.34, 0.07)	-0.02 (-0.22, 0.18)	-0.09 (-0.28, 0.10)	0.03 (-0.17, 0.23)	-0.06 (-0.27, 0.14)
**Step Time SD**	-0.08 (-0.28, 0.13)	-0.01 (-0.21, 0.19)	-0.01 (-0.21, 0.20)	-0.05 (-0.24, 0.13)	-0.03 (-0.23, 0.17)	-0.03 (-0.23, 0.16)
**Swing Time SD**	-0.0 (-0.28, 0.15)	-0.03 (-0.24, 0.18)	-0.03 (-0.25, 0.18)	-0.11 (-0.30, 0.09)	0.02 (-0.19, 0.23)	-0.03 (-0.23, 0.17)
**Stance Time SD**	0.02 (-0.18, 0.22)	0.03 (-0.17, 0.23)	-0.15 (-0.35, 0.04)	**-0.20 (-0.38, 0.01)***	-0.08 (-0.28, 0.12)	-0.15 (-0.35, 0.04)
**SST SD**	0.08 (-0.12, 0.29)	0.05 (-0.15, 0.25)	-0.03 (-0.24, 0.18)	-0.00 (-0.20, 0.19)	0.05 (-0.15, 0.26)	0.06 (-0.14, 0.26)
**DST SD**	0.08 (-0.13, 0.28)	0.06 (-0.14, 0.26)	-0.17 (-0.37, 0.03)	-0.14 (-0.33, 0.05)	**-0.22 (-0.42, -0.02)***	-0.19 (-0.38, 0.01)
**SST % of GC**	0.02 (-0.18, 0.22)	-0.03 (-0.22, 0.17)	0.12 (-0.08, 0.32)	0.12 (-0.07, 0.31)	0.13 (-0.07, 0.33)	0.15 (-0.05, 0.34)
**Swing Time % of GC**	-0.08 (-0.27, 0.13)	-0.08 (-0.28, 0.11)	0.08 (-0.11, 0.28)	0.05 (-0.13, 0.24)	0.14 (-0.06, 0.34)	0.14 (-0.06, 0.33)
**Stance Time % of GC**	0.10 (-0.09, 0.30)	0.12 (-0.07, 0.31)	-0.06 (-0.26, 0.14)	-0.04 (-0.23, 0.14)	-0.12 (-0.32, 0.08)	-0.11 (-0.30, 0.08)
**DST % of GC**	0.01 (-0.18, 0.21)	0.03 (-0.17, 0.22)	-0.02 (-0.21, 0.18)	-0.02 (-0.21, 0.16)	0.07 (-0.13, 0.27)	0.08 (-0.11, 0.27)
**DST**	-0.04 (-0.24, 0.16)	0.00 (-0.19, 0.20)	-0.08 (-0.28, 0.11)	-0.11 (-0.29, 0.08)	-0.08 (-0.27, 0.12)	-0.09 (-0.28, 0.11)
**Stride Length**	-0.12 (-0.31, 0.08)	-0.16 (-0.35, 0.03)	0.16 (-0.04, 0.36)	**0.20 (0.02, 0.38)***	**0.21 (0.02, 0.41)***	**0.24 (0.05, 0.42)***
**Step Length**	-0.16 (-0.35, 0.04)	**-0.19 (-0.38, -0.00)***	0.18 (-0.02, 0.37)	**0.20 (0.02, 0.38)***	**0.20 (0.01, 0.40)***	**0.22 (0.03, 0.41)***
**Stride Width**	0.15 (-0.04, 0.35)	**0.22 (0.03, 0.41)***	-0.09 (-0.29, 0.11)	-0.14 (-0.33, 0.04)	-0.12 (-0.32, 0.07)	-0.14 (-0.33, 0.06)

The relationships between spatiotemporal gait parameters on usual and cognitive dual-task walking and cognitive function were assessed in 102 individuals (with and without T2DM). Dual-task performance was assessed both alone and in terms of dual-task cost (percentage change from normal pace on addition of a dual-task paradigm). Linear regression was used with gait characteristic as the predictor (independent) variable and cognitive performance as the outcome (dependent variable). Results are presented as Beta Coefficients (β) with appropriate 95% Confidence Interval (95% CI). Model 1 is unadjusted whilst Model 2 adjusted for age, sex, BMI, T2DM status and education ***p<0.05, **p<0.01, ***p<0.001.**
